# BCL2A1 is associated with tumor-associated macrophages and unfavorable prognosis in human gliomas

**DOI:** 10.18632/aging.205149

**Published:** 2023-10-25

**Authors:** Lun Gao, Zhang Ye, Shu Peng, Pan Lei, Ping Song, Zhiyang Li, Long Zhou, Qiuwei Hua, Li Cheng, Hangyu Wei, Junhui Liu, Qiang Cai

**Affiliations:** 1 Department of Neurosurgery, Renmin Hospital of Wuhan University, Wuhan, China; 2 Central Laboratory, Renmin Hospital of Wuhan University, Wuhan, China; 3 School of Nursing, Kunming Medical University, Kunming, China

**Keywords:** BCL2A1, glioma, prognosis, TAMs, temozolomide

## Abstract

B-cell lymphoma 2-related protein A1 (BCL2A1) is a member of the BCL-2 family. Previous studies have shown that BCL2A1 is closely related to the tumorigenesis and resistance to chemotherapy of multiple solid tumors, such as breast cancer. However, the expression pattern and potential biological function of BCL2A1 in glioma remain unknown. For the first time, we found that the expression of BCL2A1 was higher in human glioma tissues than in normal brain tissues (NBTs) in both public datasets and an in-house cohort. High BCL2A1 expression was associated with advanced WHO grade, IDH 1/2 wild type and the mesenchymal (ME) subtype, and its overexpression in glioma predicted resistance to temozolomide (TMZ) chemotherapy and unfavorable prognosis. In addition, Gene set enrichment analysis (GSEA), Gene Ontology (GO) and Kyoto Encyclopedia of Genes and Genomes (KEGG) analysis indicated that BCL2A1 was significantly correlated with the immune response and immune-related pathways, and BCL2A1 expression was positively correlated with microenvironmental parameters (immune, stromal, and ESTIMATE scores) and macrophage infiltration. Interestingly, bioinformatic prediction and immunohistochemical/immunofluorescence staining analysis revealed that BCL2A1 expression was obviously associated with the tumor-associated macrophages (TAMs) markers CD68 and CCL2. Notably, knockdown of BCL2A1 significantly inhibited cell proliferation of U87 and U251 *in vitro*, induced smaller tumor size and prolonged survival time of mice *in vivo*.

Co-culture experiments of macrophages and GBM cells showed that BCL2A1 knockdown inhibited macrophage migration. Meanwhile, knockdown of BCL2A1 was associated with low expression of CD68 and CCL2 in intracranial xenograft model. This may suggest that BCL2A1 promotes the progression of glioma and influences the prognosis of patients by participating in TAMs infiltration. In conclusion, these findings suggest that BCL2A1 could serve as a promising prognostic indicator and immunotherapy target in gliomas.

## INTRODUCTION

Glioma is the most lethal primary malignant tumor of the central nervous system in adults, and it accounts for approximately 70% of malignant brain tumors [1]. According to the 2021 World Health Organization (WHO) classification of central nervous system tumors, glioma is categorized as WHO II–IV based on its degree of malignancy and molecular features [2]. Despite the use of advanced treatments, such as surgical resection, temozolomide (TMZ) chemotherapy, and radiation, in glioma patients, the outcomes of glioma remain unsatisfactory, especially for the outcomes of glioblastoma multiforme (GBM, WHO IV). The median survival time of GBM patients is approximately 14–16 months, and the 5-year survival rate is less than 5% [3, 4]. With the rapid development of high-throughput sequencing, some glioma-related molecular targets have been discovered. In the 2016 and 2021 revised WHO classification of central nervous system tumors, IDH1/2 status, 1p/19q, telomerase reverse transcriptase (TERT), epidermal growth factor receptor (EGFR) and human tumor protein p53 (TP53) were recommended for use in gliomas diagnosis and classification [2, 5]. However, the molecular mechanism underlying glioma is not fully understood, which hinders the development of strategies for glioma diagnosis and treatment.

In recent years, immunotherapy has become a new area of research in the field of tumor therapy, and there have been significant advances in cancer immunotherapy. Programmed cell death protein 1 (PD-1) blockade is effective in the treatment of Hodgkin’s lymphoma (HL), and cytotoxic T lymphocyte associated antigen 4 (CTLA4) immunotherapy has shown significant antitumor effects in melanoma [6]. However, immunotherapies such as CTLA4 and PD-1 blockade have not yet shown very successful clinical effects in GBM. This result may be related to the unique immune tumor microenvironment (TME) of GBM, which is composed of tumor cells, immune cells, extracellular matrix components, etc., [7]. TAMs are the most representative group of tumor-infiltrating leukocytes and the largest group of tumor-infiltrating immune cells in the immune microenvironment of glioma, consisting of tissue-resident microglia and monocyte-derived macrophages (MDM). TAMs are enriched in the glioma tumor microenvironment, and their numbers increase with increasing pathological grade, indicating the key role of TAMs in tumor development [3, 7, 8]. There is growing evidence that TAMs have multiple functions in TME of GBM, including promoting tumor growth and metastasis, angiogenesis, signal transduction, and aggravating treatment resistance [9]. Another possible factor is the lack of biomarkers to guide individual immune targets. Therefore, further elucidating the mechanism underlying tumor immune interactions and identifying new immune-related markers and therapeutic targets for glioma will help improve the prognosis of glioma patients.

The primary function of B-cell lymphoma 2 (BCL2) family proteins is to control the mitochondrial apoptosis pathway, and many BCL2 family proteins have been identified as important anticancer targets due to their important functions in cellular apoptosis [10]. BCL2A1 is a member of the BCL-2 family of anti-apoptotic proteins and one of the less well-studied anti-apoptotic BCL2 proteins. BCL2A1 has been shown to be overexpressed in many cancers and is associated with resistance to chemotherapeutic and targeted drugs [11, 12]. In addition, BCL2A1 has been identified as a biomarker for postoperative seizure control in patients with low-grade glioma (LGG, WHO grade II–III) [13]. However, the expression pattern and potential biological role of BCL2A1 in glioma remain unknown.

In this study, we explored the expression of BCL2A1 in gliomas and its relationship with glioma malignancy using public datasets and an in-house cohort. We also systematically and comprehensively evaluated the prognostic value of BCL2A1 in glioma. In addition, we investigated the relationship between BCL2A1 and immune cell infiltration and found that BCL2A1 is associated with TAMs in the TME. Immunohistochemistry (IHC) confirmed that BCL2A1 was positively correlated with the TAM markers CCL2 and CD68, and immunofluorescence (IF) showed that they colocalized in a large number of cells. Meanwhile, we demonstrated that knocking down BCL2A1 can inhibit the migration ability of co-cultured macrophages. Inhibiting the expression of BCL2A1 effectively inhibited the proliferation of tumor cells and improved the prognosis of mice *in vitro* and *in vivo*. Therefore, we speculated that BCL2A1 might accelerate tumor progression by promoting TAM infiltration in the glioma tumor microenvironment.

## MATERIALS AND METHODS

### Clinical samples

In our in-house cohort, a paraffin-embedded tissue microarray included 174 glioma and 10 NBTs. All tissue samples were obtained from the Department of Neurosurgery, Renmin Hospital of Wuhan University from January 2017 to March 2020. None of the patients received chemotherapy or radiation before surgery, and all the patients signed informed consent forms. This study was approved by the Ethics Committee of the Renmin Hospital of Wuhan University (approval number: 2012LKSZ (010) H).

### Public data acquisition and preprocessing

The RNA-Seq dataset of 33 types of cancers was downloaded from the TCGA database using UCSC Xena (https://xena.ucsc.edu/). The GlioVis database is an important platform for the visualization and analysis of brain tumors. In addition to normalized mRNA data, it also contains corresponding clinical information (http://gliovis.bioinfo.cnio.es/) [14]. In this study, a total of six datasets were utilized, including the TCGA-GBM. TCGA-LGG, TCGA-GBMLGG, CGGA, Rembrandt and Gravendeel datasets. The Oncomine (http://www.oncomine.org/) dataset was used to comprehensively analyze the expression pattern of BCL2A1 across carcinomas [15].

### Analysis of immune infiltration

The R package “ESTIMATE” was used to assess immune, stromal, and ESTIMATE scores. Tumor Immune Estimation Resource (TIMER) is a website tool for evaluating gene expression and tumor-infiltrating immune cells [16]. The Tumor Immune Single-Cell Hub (TISCH) database was used to analyze the correlations between BCL2A1 expression and infiltrating immune cells (http://tisch.comp-genomics.org/). TISCH is a scRNA-seq database focusing on the tumor microenvironment and provides detailed cell-type annotation at the single-cell level, enabling the exploration of the TME across different cancer types [17].

### Differential gene identification and enrichment analysis

Differentially expressed genes (DEGs) were identified by the R package “edgeR.”, and the cutoff criteria of DEGs were *P* value < 0.05 and |logFC| >2. DEGs were input into the STRING database to analyze their interactions (https://cn.string-db.org/). Then, PPI data were exported and uploaded to Cytoscape software 3.9.1 to display a network. The top 10 hub genes and hub network were identified by cytoHubba and MCODE plug-in Cytoscape, respectively. The DAVID dataset (https://david.ncifcrf.gov/tools.jsp) was used to conduct gene function enrichment analysis, including Gene Ontology (GO) and Kyoto Encyclopedia of Genes and Genomes (KEGG) analysis. The “clusterProfiler” package was used to perform gene set enrichment analysis (GSEA) to investigate the differences in biological pathways between samples with high and low BCL2A1 expression. The top 5 entries are shown, and the “ggplot2” package in R was used to visualize the results of GSEA.

### BCL2A1 and drug response

NCI-60 compound activity data and RNA-seq expression profiles were downloaded from the CellMiner database (http://discover.nci.nih.gov/cellminer/) to analyze the drug sensitivity of BCL2A1 [18]. Drugs approved by the FDA or clinical trials were selected for analysis, and the R packages “impute”, “limma”, “ggplot2”, and “ggpubr” were used.

### Cell culture and transfection

All cell lines (U87, U251 and THP-1) were purchased from Pricella (Wuhan, China). GBM cells were cultured in DMEM containing 10% fetal bovine serum (FBS) and human monocyte cell line THP-1 was cultured in 1640 medium containing 10% FBS at 37°C in 5% CO_2_. THP-1 cells were induced into macrophages by 100 ng/mL concentration of PMA for subsequent co-culture experiments. BCL2A1 and the control plasmid were purchased from the GENE (Shanghai, China). BCL2A1-shRNA and control plasmid were transfected into GBM cells with lipo3000 (Invitrogen, USA) according to the manufacturer’s instructions.

### CCK8 assay

Cells were implanted into 96 Wells at a density of 5000/well. CCK8 reagent (Dojindo, Kumamoto, Japan) added at 0 h, 24 h, 48 h, 72 h, and 96 h, and the absorbance at 450 nm was measured using a Microplate System (Olympus, Tokyo, Japan).

### Migration assay of co-cultured macrophages

THP-1 cells were induced into macrophages using PMA, and then 40,000 macrophages were planted into Transwell’s upper chamber and 100,000 BCL2A1 knockdown and control GBM cells were planted into lower chamber. GBM and macrophages were co-cultured for 24 h and fixed with 4% paraformaldehyde, stained with crystal violet, air-dried and photographed under an inverted microscope (Olympus, Tokyo, Japan).

### Western blot

PIRA lysate was added to the cells, lysed on ice for 30 minutes, sonicated and centrifuged, and boiled in protein loading buffer. Equal amounts of protein were then added to SDS-PAGE gels and subsequently transferred to PVDF (Millipore, USA) membranes. Subsequently, membranes were blocked and incubated with anti-GAPDH (Proteintech, Wuhan, China, 1:5000) and anti-BCL2A1 (Abways, Shanghai, China, 1:1000) overnight. The following day, the membranes incubated with secondary antibodies and visualized with ChemiDoc™ Touch Imaging System (Bio-Rad, USA).

### Intracranial xenograft model

Balb/cA-nu mice purchased from Shaulaibao Biotechnology Co., Ltd (Wuhan, China) were fed adaptively in SPF environment for one week, and then 1 × 10^6^ U87-Nc-BCL2A1 or U87-Sh-BCL2A1 cells were injected into the right striatum of mice to establish intracranial xenograft model. The mice were monitored daily and killed when they developed severe neurological symptoms or weight loss. The brains of mice were collected and embedded in paraffin for immunohistochemistry and Hype staining. All animal experiments have been examined and approved by the Animal Welfare Ethics Committee of Renmin Hospital of Wuhan University.

### Immunohistochemical staining

Paraffin sections were dewaxed with xylene (3 times) and different concentrations of ethanol (100%, 95%, 75%) and then washed with PBS buffer three times. Sodium citrate (10 mM, pH 6.0) was used for antigen retrieval, and the samples were incubated for 10 minutes at 100°C. The sections were incubated with 3% H_2_O_2_ for 10 minutes to remove endogenous peroxidase and blocked with 1% bovine serum albumin (BSA) for 30 minutes. Primary antibody was added to the paraffin sections and incubated at 4°C overnight. The next day, HRP-conjugated goat anti-rabbit IgG (GB23303, Servicebio, Wuhan, China) secondary antibody was added and incubated for 1 hour. 3,3′-Diaminobenzidine (DAB) (GDP1061, Servicebio) reagent was added to the paraffin sections, observed under a microscope, and washed with running water to terminate the reaction. The sections were stained with hematoxylin for one minute, sealed with a neutral resin and visualized under the Pannoramic Scanner (3DHISTECH, Budapest, Hungary). The immune score was determined according to the proportion and intensity of positive cells. The results were scored as follows: 0, no staining; 1, weak staining; 2, moderate staining; and 3, strong staining. The score according to the proportion of positive cells is as follows: 0, <10%; 1, 10–25%; 2, 26–50%; 3, 51–75%; and 4, >75%. The final immune score was calculated with the following formula: staining intensity score *x* positive cell number score. We defined 0–4 as low expression and 6–12 as high expression. Immunohistochemical (IHC) staining results were analyzed independently by three individuals.

### Immunofluorescence staining

After dewaxing and removal of endogenous peroxidase, paraffin sections were blocked with 1% BSA for 30 min. Antibodies against BCL2A1 (CY5582, Abways), CCL2 (GB11199, Servicebio) and CD68 (GB113150, Servicebio) were added in turn and incubated overnight. Then, HRP-conjugated goat anti-rabbit IgG secondary antibody and tyramide signal amplification (TSA) (TSA-488, TSA-555, TSA-547, G1236, Servicebio) were added and incubated. Then, the cell nuclei were stained with 4’,6-diamidino-2-phenylindole dihydrochloride (DAPI), and the results were visualized with a Pannoramic Scanner (3DHISTECH).

### Statistical analysis

Data are presented as the means ± standard deviations (SD). Student’s *t* test was used to analyze differences between two groups, and one-way ANOVA was employed to compare differences among multiple groups. Spearman correlation analysis was utilized to investigate the correlation between parameters. The BCL2A1 high and low expression groups were determined according to gene expression level relative to the given optimal cutoff value. Kaplan–Meier survival analysis was used to compare the overall survival (OS) of glioma patients. In this study, statistical analysis was performed using GraphPad 8 (version 8.0) and R software (version 4.2). A *P* value of less than 0.05 was considered significant.

### Data availability statements

The data generated in the present study may be requested from the corresponding author.

## RESULTS

### BCL2A1 was overexpressed in human gliomas

**Figure 1:**
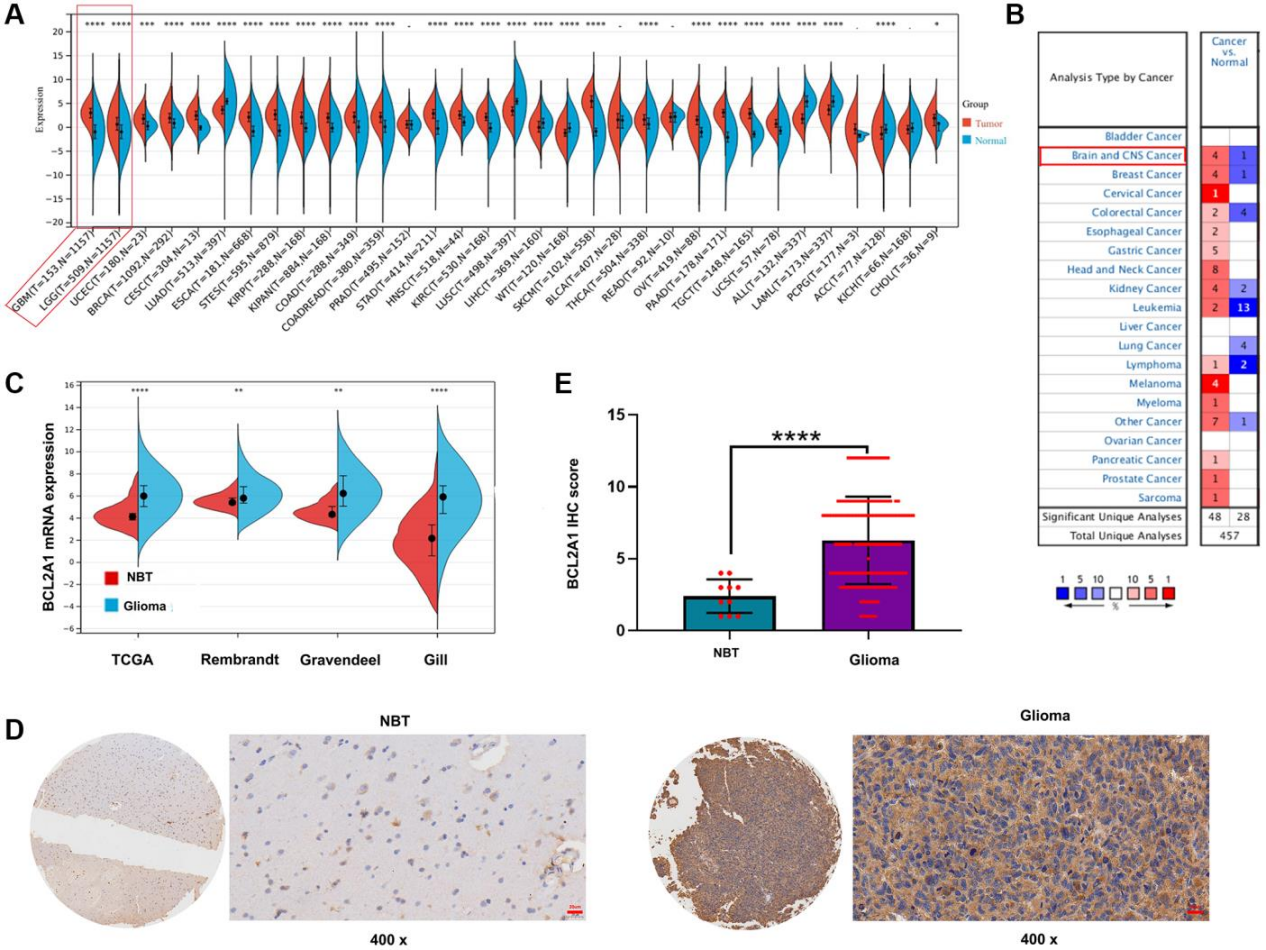
**BCL2A1 was overexpressed in gliomas.** (**A**) The expression of BCL2A1 in 33 human cancers and normal tissues in TCGA and GTEx databases. (**B**) The expression of BCL2A1 in different types of cancers in Oncomine. (**C**) The mRNA expression of BCL2A1 in gliomas and NBTs in public datasets. (**D**, **E**) IHC staining analysis of BCL2A1 in glioma NBTs in an in-house cohort. ^*^P < 0.05, ^**^P < 0.01. ^***^P < 0.001, ^****^P < 0.0001. Abbreviations: IHC: immunohistochemistry; NBTs: normal brain tissues.

To investigate BCL2A1 mRNA expression across cancers, we downloaded RNA-seq data for 33 types of tumor tissues and normal tissues from the TCGA and GTEx datasets. The results demonstrated that BCL2A1 was significantly upregulated in most tumors, including GBM and LGG, and downregulated in lung adenocarcinoma (LUAD), lung squamous cell carcinoma (LUSC), liver hepatocellular carcinoma (LIHC), Wilms tumor (WT), acute myeloid leukemia (LAML) and acute lymphoblastic leukemia (ALL). In addition, there was no significant difference between prostate adenocarcinoma (PRAD), bladder urothelial carcinoma (BLCA), rectum adenocarcinoma (READ), ovarian serous cystadenocarcinoma (OV), and cholangiocarcinoma (CHOL) and the corresponding normal tissues (Figure 1A). Analysis of BCL2A1 in the Oncomine database showed that BCL2A1 expression was higher in brain and CNS cancer tissues than in normal tissues (Figure 1B).

In addition, we used TCGA, Gill, Rembrandt and Gravendeel datasets to further investigate the BCL2A1 expression pattern in human gliomas. The results demonstrated that BCL2A1 expression was higher in gliomas than in NBTs (Figure 1C). IHC of an in-house cohort consisting of 10 NBTs and 174 glioma samples also confirmed that BCL2A1 had a higher expression level in glioma tissues (Figure 1D, 1E).

### BCL2A1 was associated with glioma malignancy

**Figure 2:**
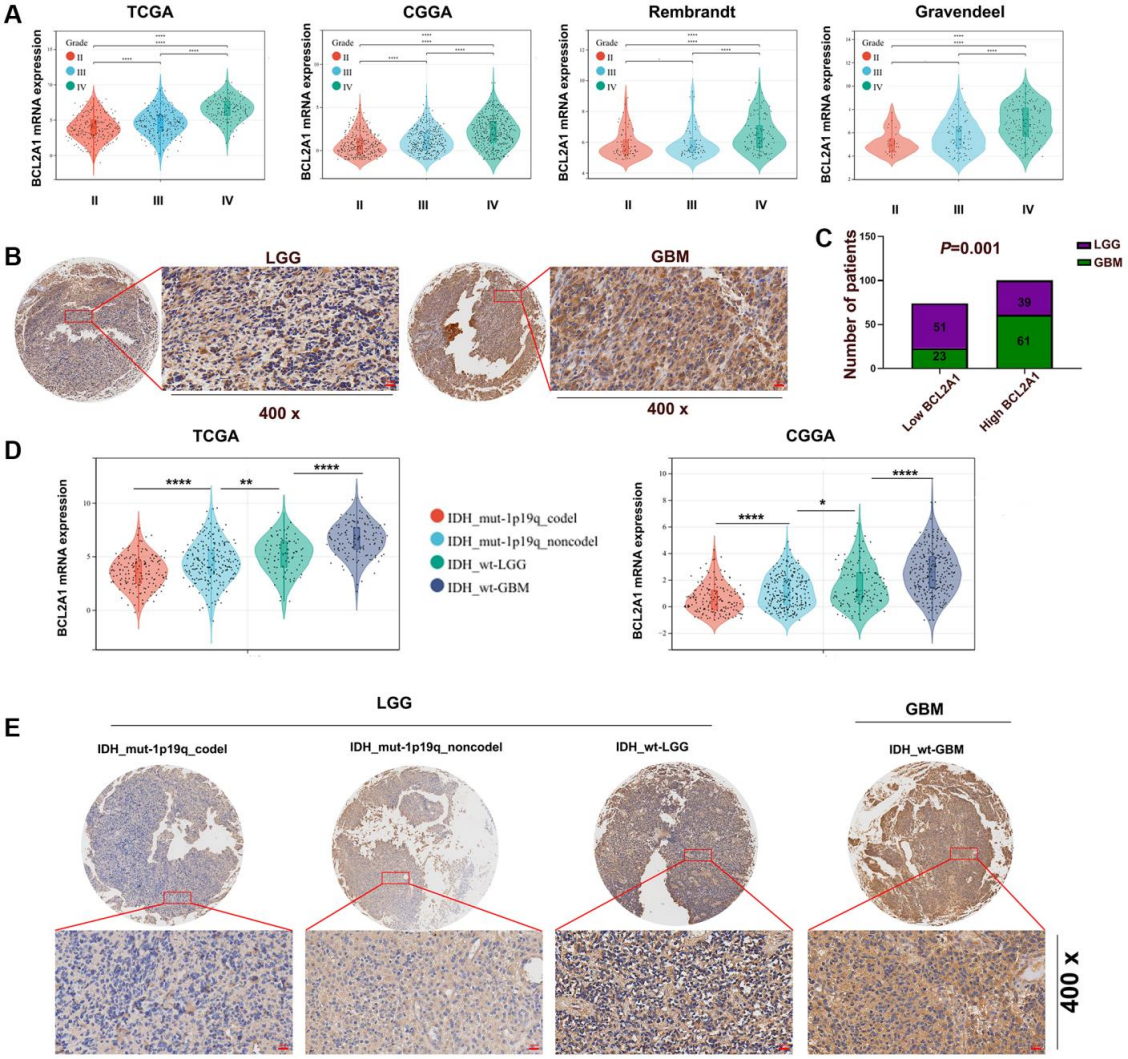
**BCL2A1 was associated with the malignancy of gliomas.** (**A**) TCGA, CGGA, Rembrandt and Gravendeel datasets were utilized to assess the expression of BCL2A1 in tumors of different grades. (**B**, **C**) IHC staining of BCL2A1 in LGG and GBM tissues. (**D**) BCL2A1 expression in gliomas with different IDH and 1p19q statuses in TCGA and CGGA datasets. (**E**) The expression of BCL2A1 in gliomas with different IDH and 1p19q statuses was analyzed by immunohistochemistry. Abbreviations: mut: mutation; WT: wild type; codel: codeletion; non-codel: non-codeletion. ^*^P < 0.05, ^**^P < 0.01. ^***^P < 0.001, ^****^P < 0.0001.

Glioma was classified as grades II-IV according to the degree of malignancy, and public TCGA, CGGA, Rembrandt and Gravdendeel datasets indicated that BCL2A1 expression increased with increasing glioma grade (Figure 2A). In our in-house cohort, IHC staining analysis demonstrated that BCL2A1 expression was higher in GBM tissues than in LGG tissues (Figure 2B, 2C). IDH 1/2 and 1p19q mutation statuses are widely used in the diagnosis and classification of glioma. Glioma patients with IDH 1/2 wild type (WT) and chromosome 1p/19q non-codeletion have a worse prognosis [19]. In public datasets and an in-house cohort, BCL2A1 expression was higher in GBM with IDH1/2 wild type than in LGG with or without IDH mutation (Figure 2D, 2E). The correlation between BCL2A1 and clinicopathological characteristics in patients with gliomas in the in-house cohort, TCGA and CGGA is presented in Tables 1, 2 and Supplementary Table 1, respectively.

**Table 1:** Correlation between BCL2A1 and clinicopathological characteristics in patients with gliomas in in-house cohort.

**Clinicopathological characteristics**	**BCL2A1 expression**		***P* value**
	**Low (*n* = 74)**	**High (*n* = 100)**	
**Age**			
≥60	22	29	>0.05
<60	52	71	
**Gender**			
Male	38	47	>0.05
Female	36	53	
**WHO grade**			
II–III	51	39	0.001
IV	23	61	
**IDH status**			
Mutant	45	37	0.002
Wild-type	29	63	
**KPS**			
≥80	47	62	>0.05
<80	27	38	

**Table 2:** Correlation between BCL2A1 and clinicopathological characteristics in patients with gliomas in TCGA.

**Clinicopathological characteristics**	**BCL2A1 expression**		***P* value**
	**Low (*n* = 333)**	**High (*n* = 333)**	
**Age**			
≥60	43	103	<0.001
<60	252	210	
**Gender**			
Male	167	187	<0.001
Female	128	126	
**WHO grade**			
II–III	283	186	<0.001
IV	18	131	
**IDH status**			
Mutant	281	146	<0.001
Wild-type	50	182	
**Chr.1p19q**			
Codeletion	131	37	<0.001
Non-codeletion	200	292	
**MGMT promoter**			
Methylation	282	194	<0.001
Unmethylation	50	109	
**TERT status**			
Mutant	86	68	<0.05
Wild-type	96	70	
**ATRX status**			
Mutant	111	84	<0.05
Wild-type	220	241	

### BCL2A1 was associated with GBM of mesenchymal subtype

**Figure 3:**
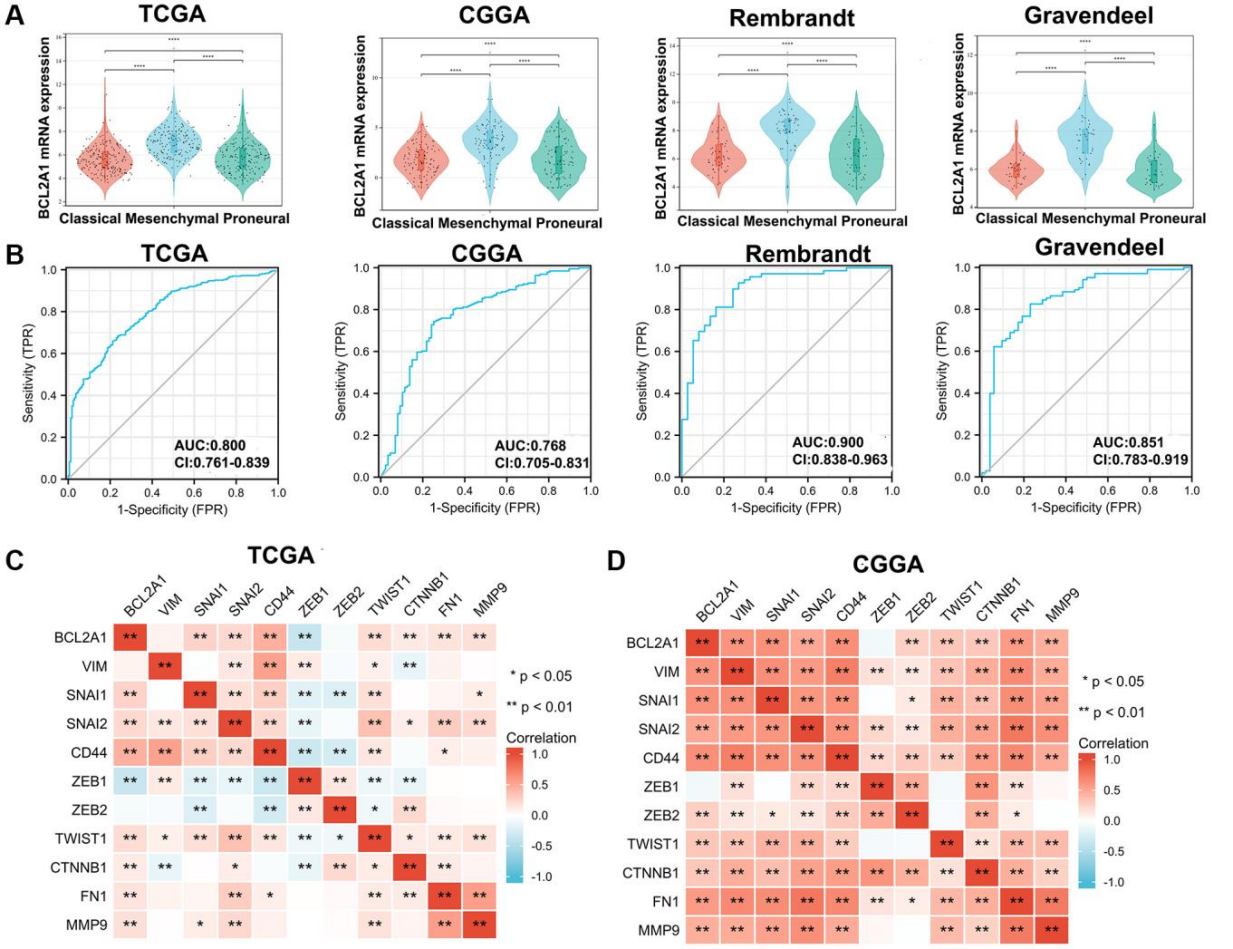
**BCL2A1 was associated with GBM of the mesenchymal subtype.** (**A**) TCGA, CGGA, Rembrandt and Gravendeel datasets were used to investigate the expression of BCL2A1 in GBM with different subtypes. (**B**) Accuracy of BCL2A1 in predicting the mesenchymal subtype as determined using ROC curves. (**C**, **D**) The Spearman correlation method was used to explore the relationship between BCL2A1 and mesenchymal-related markers in TCGA and CGGA. Abbreviations: AUC: area under the curve; ROC: receiver operating characteristic. ^*^P < 0.05, ^**^P < 0.01, ^****^P < 0.0001.

Philips et al. classified GBM into three molecular subclasses, named the proneural, mesenchymal (ME), and classical subtypes. GBM patients with the mesenchymal subtype have a poor prognosis, and patients with recurrence are more likely to convert to the mesenchymal phenotype [20]. TCGA, CGGA, Rembrandt and Gravendeel were utilized to explore differences in BCL2A1 expression among GBM subtypes, and the results confirmed that BCL2A1 expression was enriched in the mesenchymal subtype (Figure 3A). The receiver-operating characteristic (ROC) curve was used to evaluate the specificity of BCL2A1 expression in the mesenchymal subtype of GBM. As expected, acceptable area under the curve (AUC) values of up to 0.800, 0.768, 0.900 and 0.851 in the TCGA, CGGA, Rembrandt and Gravendeel datasets, respectively, were observed (Figure 3B). In addition, the correlation of BCL2A1 expression and mesenchymal-related gene expression was analyzed, and the results showed that BCL2A1 expression was positively correlated with the expression of GBM mesenchymal subtype markers in the TCGA and CGGA datasets (Figure 3C, 3D). These results suggested that BCL2A1 may be a predictive biomarker for the GBM mesenchymal subtype.

### BCL2A1 is a novel prognostic marker for glioma patients

**Figure 4:**
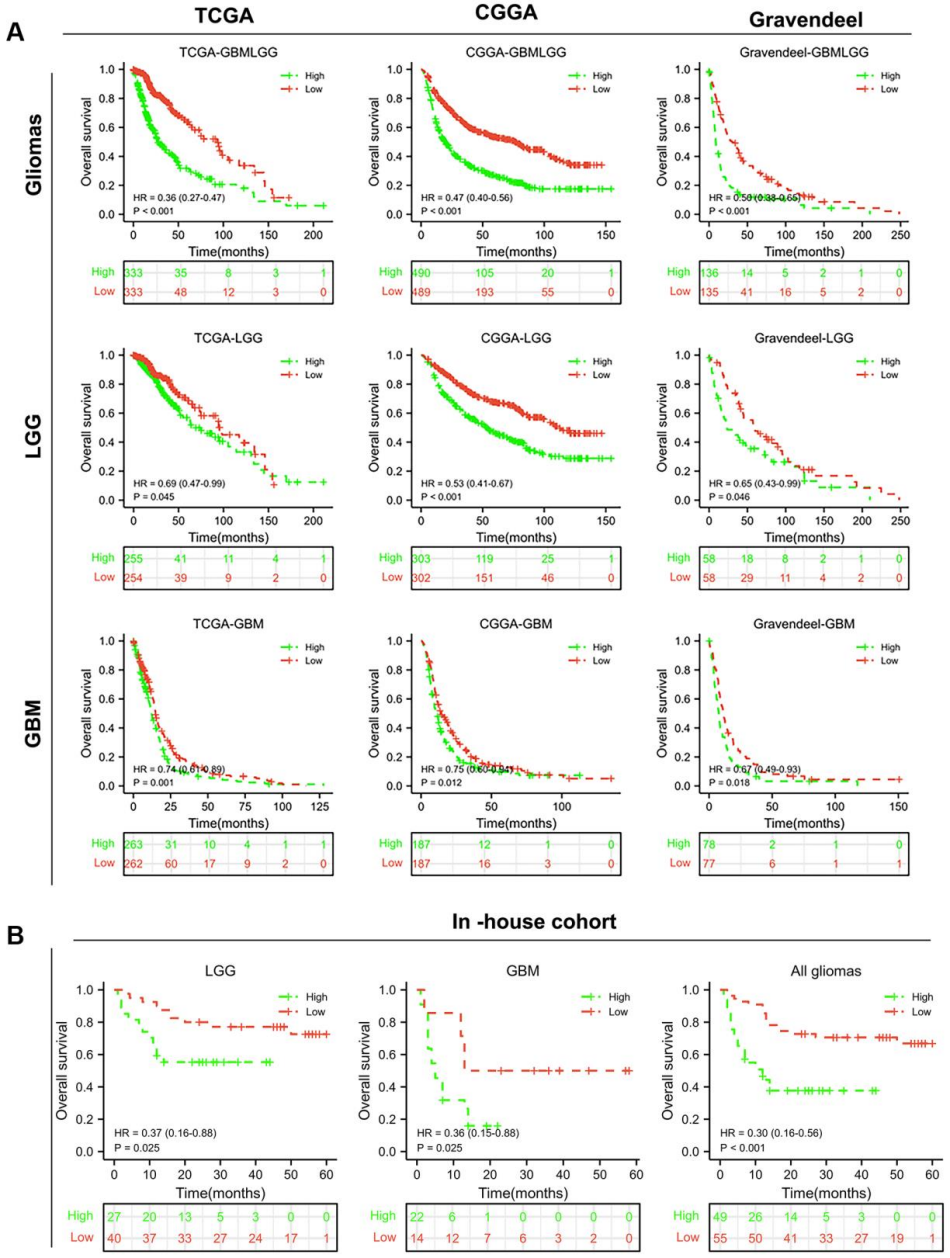
**BCL2A1 was an independent prognostic factor for gliomas.** (**A**) Kaplan–Meier survival curves of BCL2A1 in TCGA, CGGA and Gravendeel. (**B**) Prognostic value of BCL2A1 in an in-house cohort. Abbreviation: HR, hazard ratio.

To evaluate the prognostic value of BCL2A1 in glioma patients, Kaplan-Meier curve analyses were performed based on TCGA, CGGA and Rembrandt datasets. The results confirmed that glioma patients with high BCL2A1 expression had significantly shorter overall survival than glioma patients with low BCL2A1 expression. Interestingly, both GBM and LGG patients with high BCL2A1 expression had a worse prognosis than patients with low BCL2A1 expression in public datasets (Figure 4A). To further verify these results, we performed a Kaplan-Meier survival analysis in our in-house cohort. As we hypothesized, high expression of BCL2A1 reduced survival time in all glioma patients, including LGG and GBM (Figure 4B).

### Gene functional enrichment analysis and construction of a protein–protein interaction network

**Figure 5:**
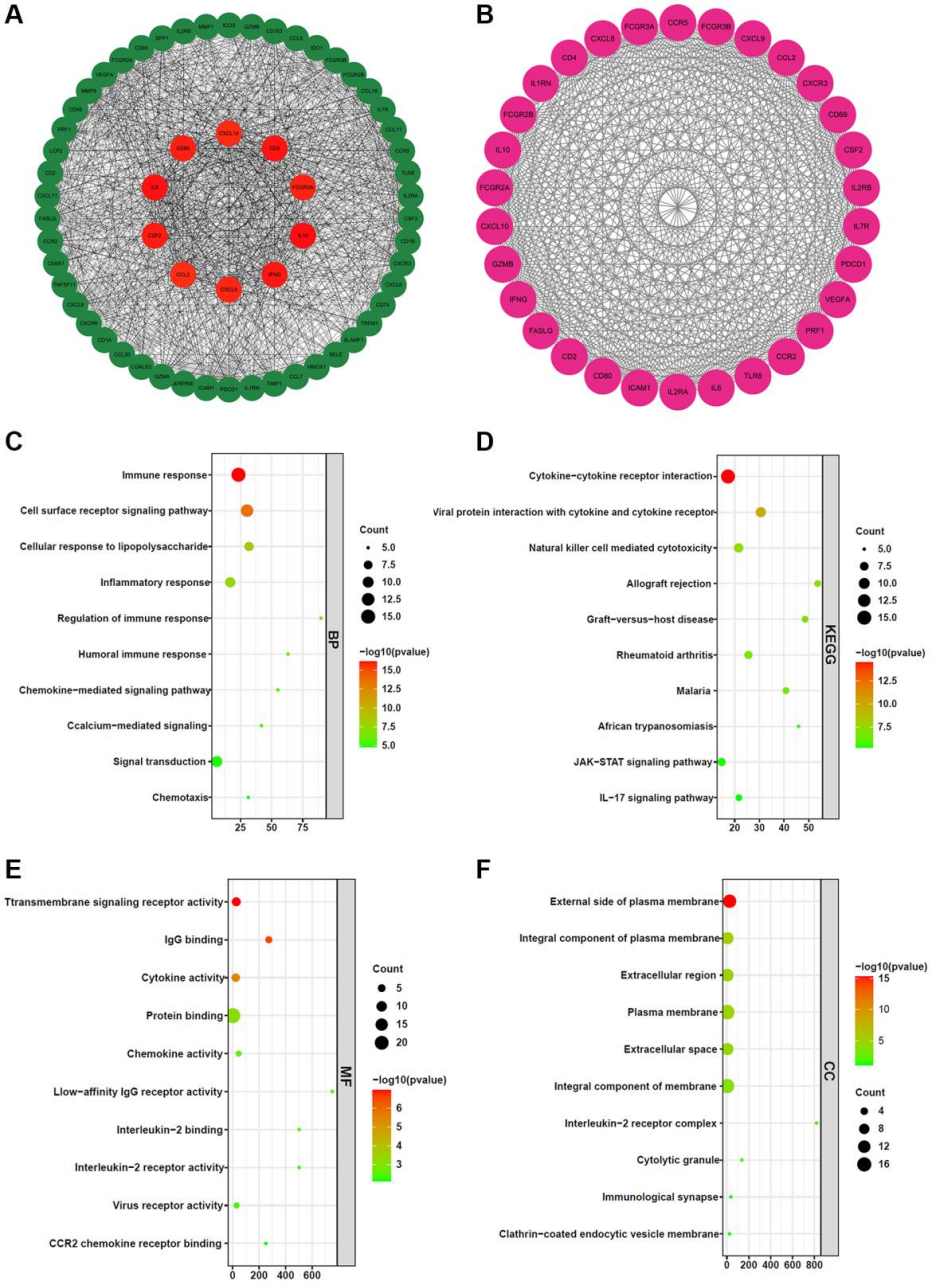
**Gene functional enrichment analysis and construction of the protein–protein interaction network.** (**A**) The top 60 hub genes of the PPI network. (**B**) Key network module was screened by MCDOE plug-in Cytoscape software. (**C**) Biological process (BP). (**D**) KEGG pathways. (**E**) Molecular function (MF). (**F**) Cellular component (CC).

To investigate the biological function of BCL2A1 in gliomas, we screened 14 downregulated genes and 510 upregulated genes between the high and low BCL2A1 expression groups in TCGA-GBMLGG. The top 60 hub genes were identified by the cytoHubba plug-in Cytoscape, and the corresponding protein–protein interaction (PPI) network is shown in Figure 5A. The top 10 hub genes were IL10, IFNG, CXCL9, CCL2, CSF2, IL6, CD80, CXCL10, CD4 and FCGR3A (red). In addition, we identified key network module genes of the DEGs by the MCDOE plug-in Cytoscape (Figure 5B), and gene functional enrichment analysis was performed on these genes using the DAVID database. Notably, biological process (BP) results showed that BCL2A1 was involved in the immune response, cell surface receptor signaling pathway, cellular response to lipopolysaccharide, regulation of immune response, inflammatory response, humoral immune response, and so on (Figure 5C). For KEGG, these genes were enriched in cytokine-cytokine receptor interaction, viral protein interaction with cytokine and cytokine receptor, natural killer cell mediated cytotoxicity, IL17 signaling pathway and so on (Figure 5D). For molecular functions (MF), these genes were significantly related to transmembrane signaling receptor activity, IgG binding, cytokine activity, chemokines, protein binding and so on (Figure 5E). For the cellular component (CC), these genes were associated with the external side of the plasma membrane, integral component of the plasma membrane, extracellular region, plasma membrane, immunological synapse and so on (Figure 5F).

**Figure 6:**
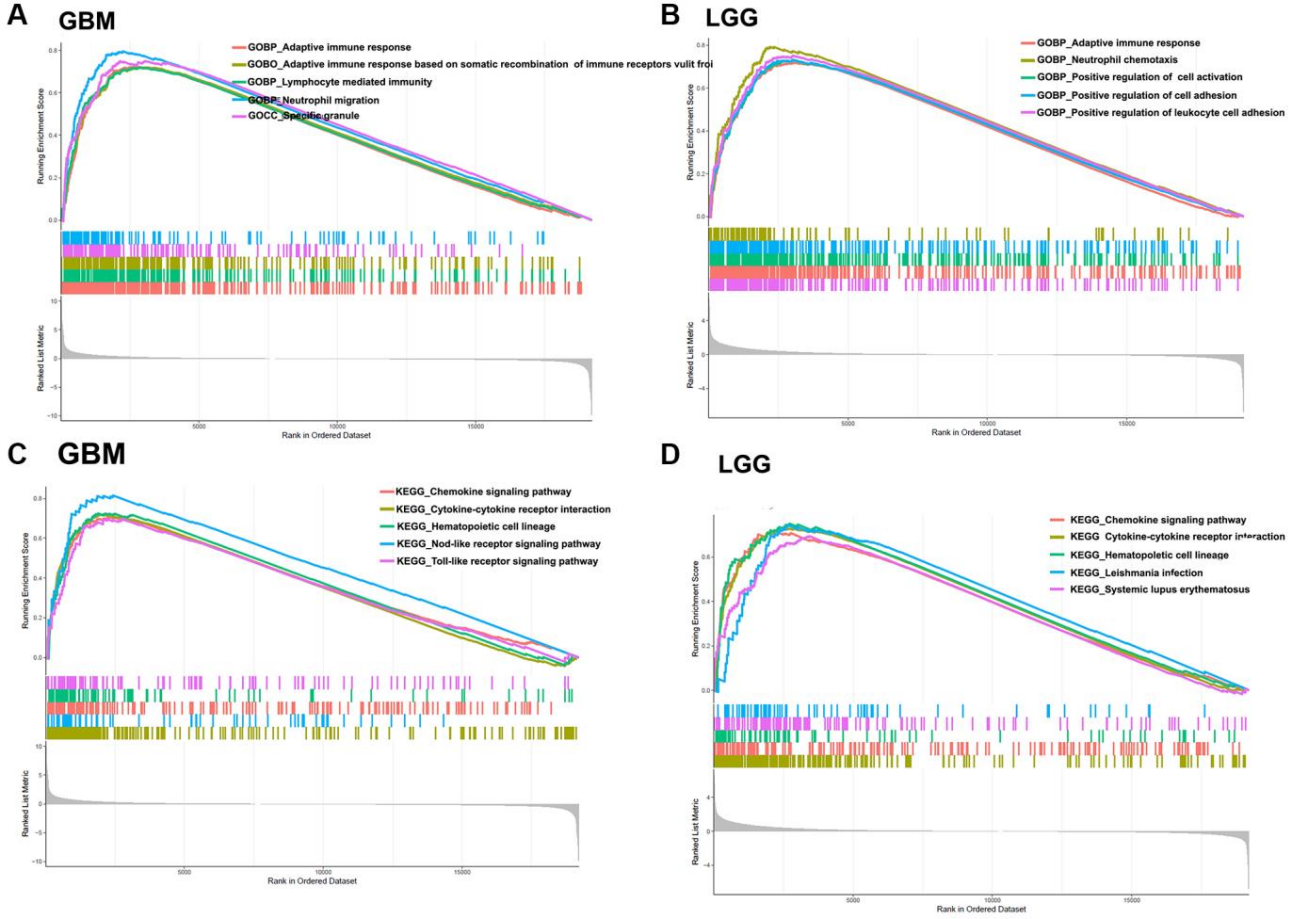
**GSEA.** (**A**) Gene Ontology (GO) analysis in GBM. (**B**) GO analysis in LGG. (**C**) KEGG analysis in GBM. (**D**) KEGG analysis in LGG.

GSEA enrichment analysis is another effective method to investigate the biological function of BCL2A1. Gene Ontology (GO) analysis showed that BCL2A1 was associated with the adaptive immune response, neutrophil chemotaxis, response to chemotaxis, neutrophil migration and so on in GBM and LGG. (Figure 6A, 6B, Supplementary Tables 2, 3). KEGG analysis confirmed that BCL2A1 was related to cytokine-cytokine receptor interactions and the chemokine signaling pathway. Toll-like receptor signaling pathway, natural killer cell-mediated cytotoxicity, T cell receptor signaling pathway, and B cell receptor signaling pathway in both GBM and LGG (Figure 6C, 6D, Supplementary Table 4, 5). These results suggested that BCL2A1 may be involved in the glioma immune response.

### BCL2A1 was associated with immune infiltration and immune-related markers in gliomas

**Figure 7:**
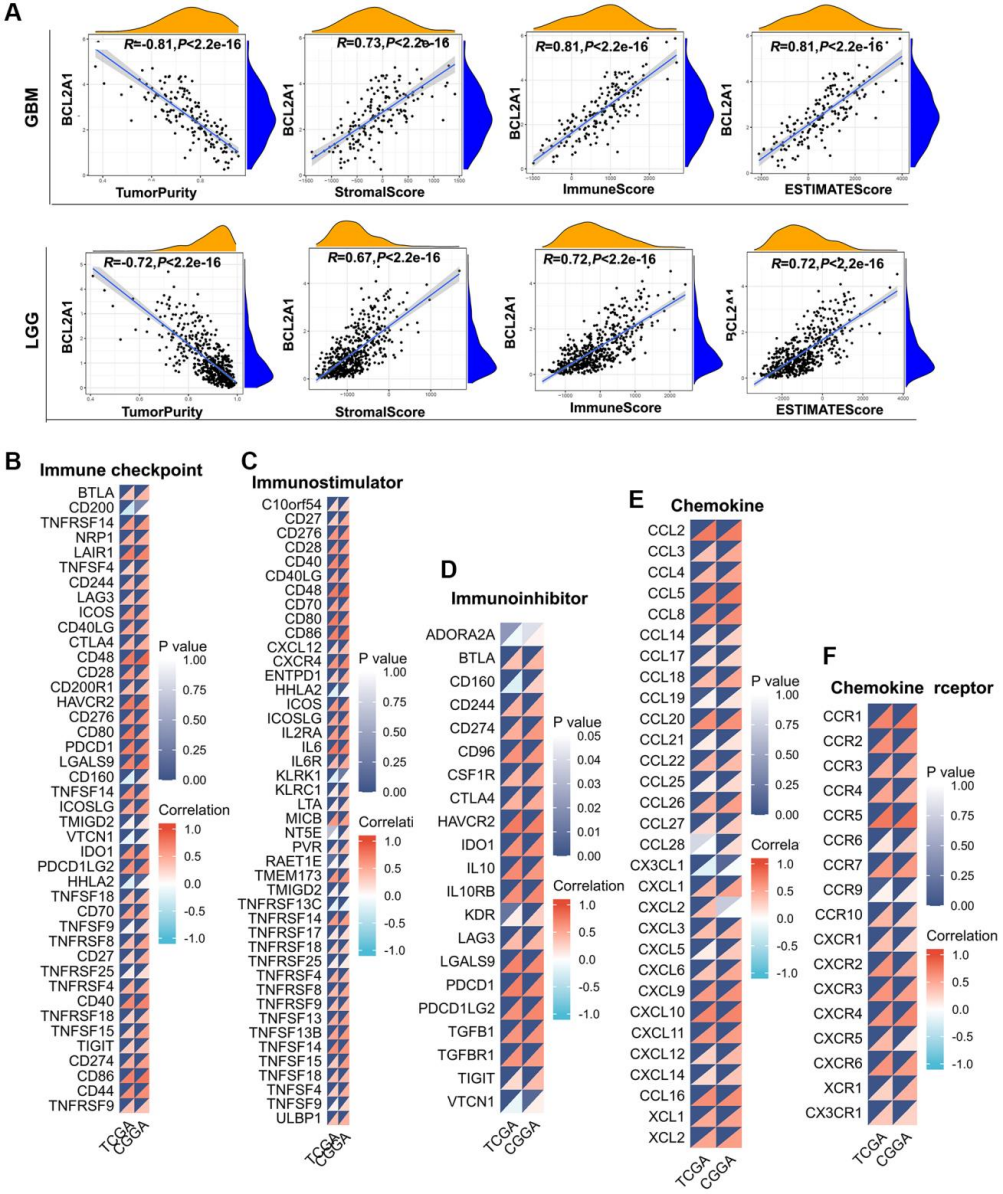
**BCL2A1 was associated with immune infiltration and immune-related markers in gliomas.** (**A**) Correlation between BCL2A1 expression and tumor purity, stromal score, immune score and ESTIMATE score in GBM and LGG. (**B**) Correlation between BCL2A1 expression and immune checkpoint gene expression. (**C**) Correlation between BCL2A1 expression and immune activation gene expression. (**D**) Correlation between BCL2A1 and immunosuppressive genes. (**E**) Correlation between BCL2A1 and chemokines. (**F**) Correlation between BCL2A1 and chemokine receptors.

The TME plays a significant role in tumorigenesis and progression, and immune and stromal cells are the two main types of nontumor cells that influence the prognosis of patients [21]. The ESTIMATE algorithm was used to assess the correlation of BCL2A1 with the TME and immune cell infiltration. The results showed that BCL2A1 was positively correlated with the immune, stromal, and ESTIMATE scores in GBM and LGG (Figure 7A). Based on the above results, we speculated that BCL2A1 might be involved in the immune regulation of glioma. Hence, we performed correlation analyses to assess the relationship of BCL2A1 with immune-related genes. Strikingly, almost all genes associated with immune checkpoints, immunological stimulation, immunosuppression, chemokines, and chemokine receptors were positively related to BCL2A1 (Figure 7B–7F).

To further understand the effects of BCL2A1 on immune regulation, we analyzed the correlation between BCL2A1 and a series of immune cell markers in TCGA and CGGA datasets. To characterize immune cells in gliomas, the immune-related genes presented in Table 3 were analyzed. The results suggested that BCL2A1 was positively correlated with most of the immune markers in various immune cells.

**Table 3:** Correlation between GMFG expression and markers of immune cells in TCGA and CGGA datasets.

	**Biomarker**	**TCGA**		**CGGA**	
		**Correlation**	***P*-value**	**Correlation**	***P*-value**
**CD8+ T-cell**	CD8A	0.4	<0.001	0.51	<0.001
	CD8B	0.3	<0.001	0.58	<0.001
**T-cell**	CD3D	0.64	<0.001	0.64	<0.001
	CD3E	0.65	<0.001	0.71	<0.001
**B cell**	CD79A	0.37	<0.001	0.45	<0.001
	CD19	0.35	<0.001	0.4	<0.001
**Monocyte**	CD86	0.71	<0.001	0.77	<0.001
**TAM**	CD68	0.75	<0.001	0.78	<0.001
	CCL2	0.75	<0.001	0.74	<0.001
	IL10	0.67	<0.001	0.6	<0.001
**M1 macrophage**	NOS2	−0.02	0.61	0.06	0.04
	IRF5	0.55	<0.001	0.57	<0.001
	CD80	0.75	<0.001	0.64	<0.001
**M2 macrophage**	CD163	0.66	<0.001	0.68	<0.001
	MS4A4A	0.68	<0.001	0.74	<0.001
	MSR1	0.8	<0.001	0.75	<0.001
**Neutrophil**	ITGAM	0.56	<0.001	0.58	<0.001
	CCR7	0.59	<0.001	0.58	<0.001
**Natural killer cell**	KIR2DL1	−	−	0.08	0.05
	KIR2DL3	−	−	0.14	<0.001
	KIR2DL4	−	−	0.38	<0.001
	KIR3DL1	−	−	0.07	0.08
	KIR3DL2	−	−	0.11	0.004
	KIR3DL3	−	−	-0.06	0.12
	KIR2DS4	−	−	0.19	<0.001
**Dendritic cell**	HLA-DPB1	0.62	<0.001	−	−
	HLA-DQB1	0.33	<0.001	−	−
	HLA-DRA	0.72	<0.001	−	−
	HLA-DPA1	0.68	<0.001	−	−
	CD11c	0.6	<0.001	0.52	<0.001

### BCL2A1 was associated with macrophages and monocytes in gliomas

**Figure 8:**
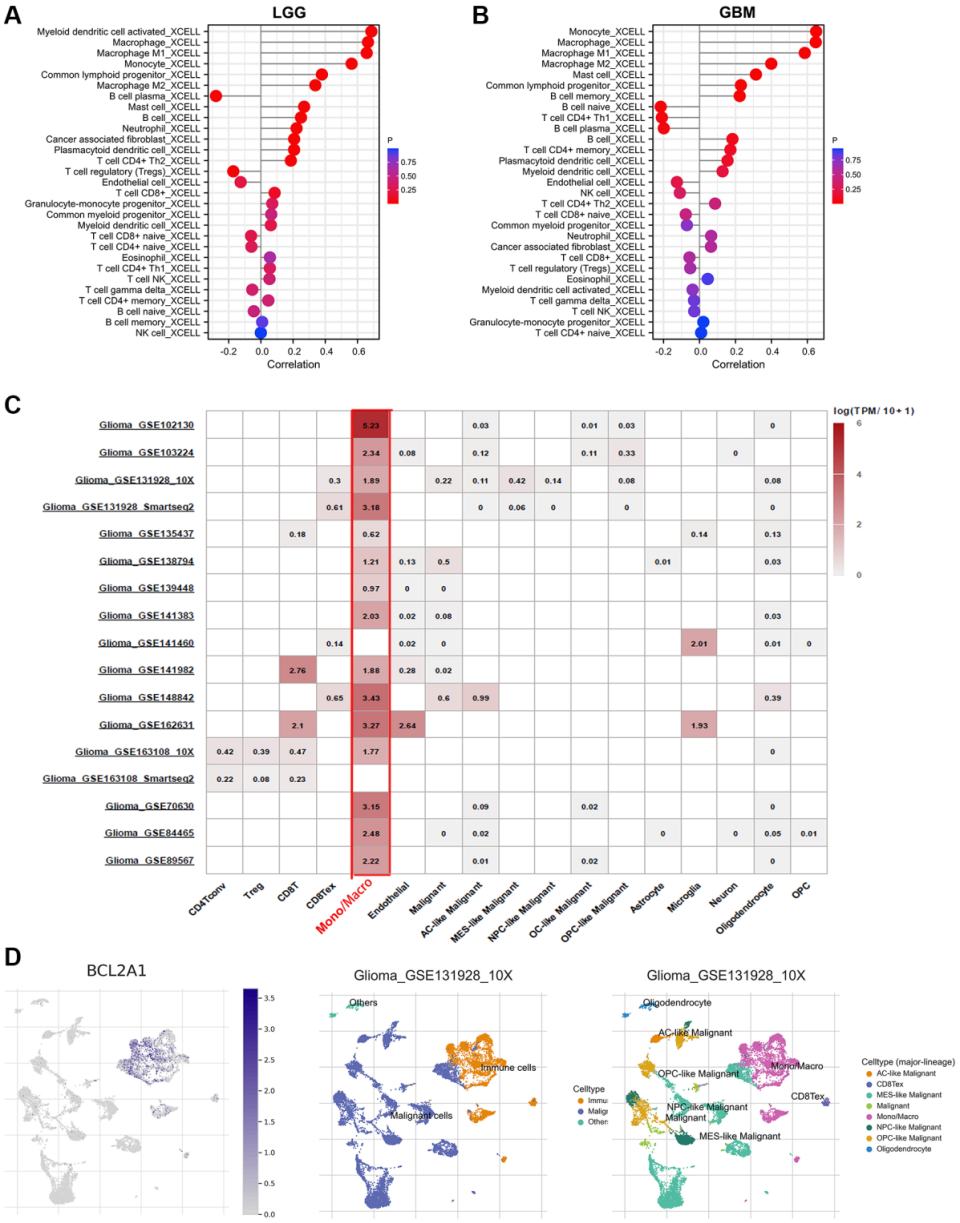
**BCL2A1 was associated with macrophages and monocytes in gliomas.** (**A**) Correlation between BCL2A1 expression and 29 infiltrating immune cells in LGG based on the TIMER 2.0 database. (**B**) Correlation between BCL2A1 expression and 29 infiltrating immune cells in GBM based on the TIMER 2.0 database. (**C**) BCL2A1 expression in 17 glioma single-cell clusters. (**D**) UMAP plot showing BCL2A1 and cell type.

The type and number of immune cells in the tumor microenvironment are related to tumor development and immunotherapy [22]. Therefore, we utilized the TIMER 2.0 dataset to evaluate the correlation between BCL2A1 expression and immune cell infiltration levels in glioma. The results showed that the expression level of BCL2A1 was positively correlated with macrophages, monocytes and B cells in GBM and LGG (Figure 8A, 8B). In addition, single-cell sequencing data from the TISCH2.0 database were used to further investigate the relationship between BCL2A1 and immune infiltration in gliomas. We found that 15 of 17 glioma single-cell sequencing datasets showed that BCL2A1 expression was mainly associated with the infiltration of macrophages and monocytes in the TISCH 2.0 database (Figure 8C, 8D and Supplementary Figure 1).

### BCL2A1 was associated with tumor-associated macrophage infiltration in gliomas

**Figure 9:**
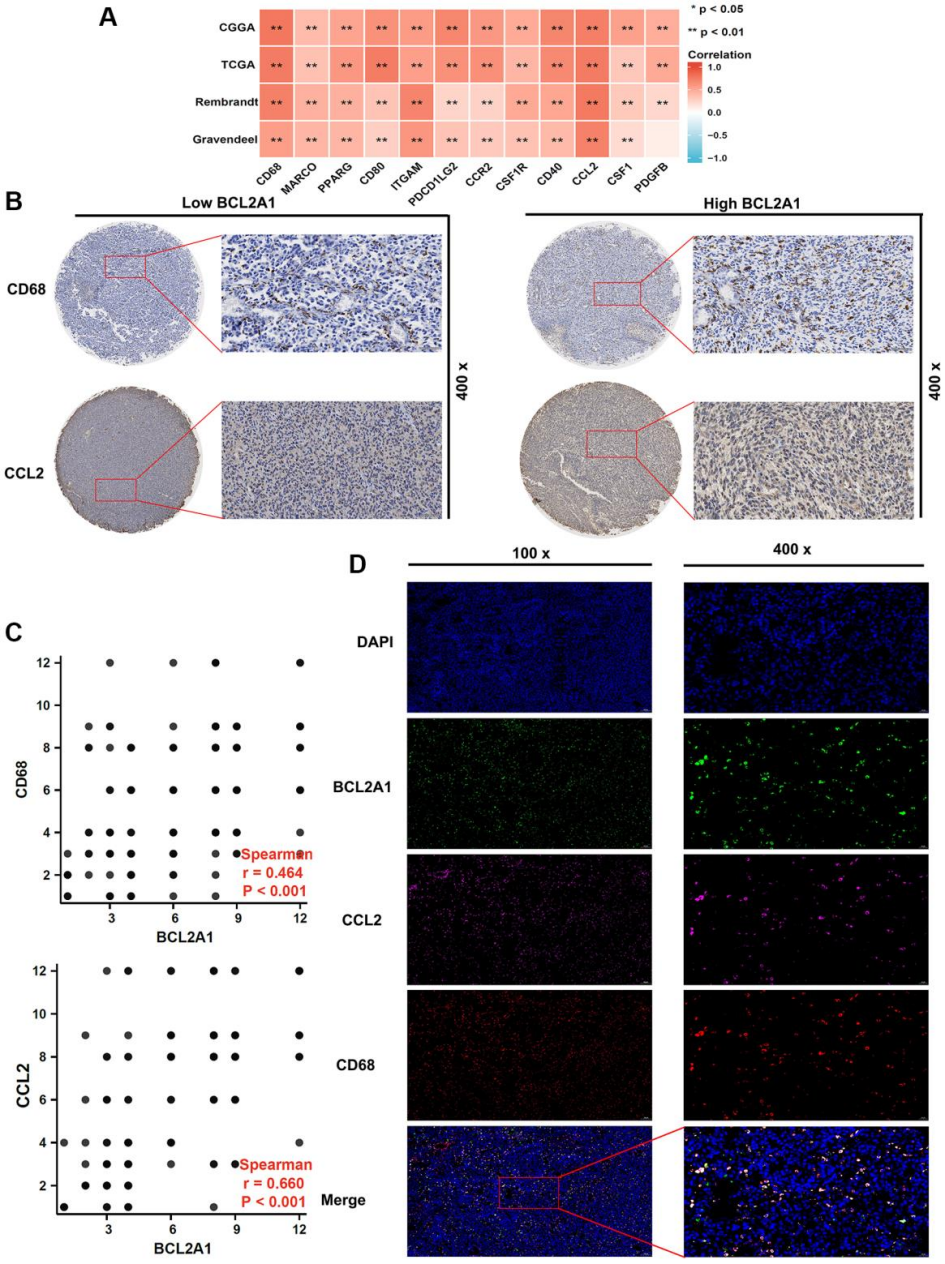
**BCL2A1 was associated with tumor-associated macrophage infiltration in gliomas.** (**A**) The correlation between BCL2A1 and tumor-associated macrophage markers in TCGA, CGGA, Rembrandt and Gravendeel datasets. (**B**) IHC staining analysis of CD68 and CCL2 in glioma tissues. (**C**) Spearman correlation was used to explore the correlation between TAM infiltration and BCL2A1 expression. (**D**) The relationship between BCL2A1, CD68 and CCL2 was analyzed by multiple immunofluorescences. Abbreviation: TAMs: tumor-associated macrophages.

Tumor-associated macrophages (TAMs) account for 50% of the total noncancer cell population in glioma and promote the proliferation, survival and migration of glioma cells [23]. Therefore, we investigated the association of BCL2A1 with tumor-associated macrophage markers through the TCGA, CGGA, Rembrandt and Gravendeel databases. The results confirmed that BCL2A1 was positively correlated with this series of tumor-related macrophage markers, especially CCL2 and CD68 (Figure 9A). Then, we performed IHC staining analysis for CD68 and CCL2 in an in-house cohort, and the results showed that high BCL2A1 expression was significantly associated with CD68 and CCL2 expression in gliomas (Figure 9B, 9C). Moreover, multiple fluorescence staining showed that BCL2A1, CD68 and CCL2 were coexpressed in glioma tissues (Figure 9D). Therefore, we speculate that BCL2A1 is closely associated with tumor-associated macrophage infiltration in gliomas.

### BCL2A1 was an independent predictor of response to temozolomide in gliomas

**Figure 10:**
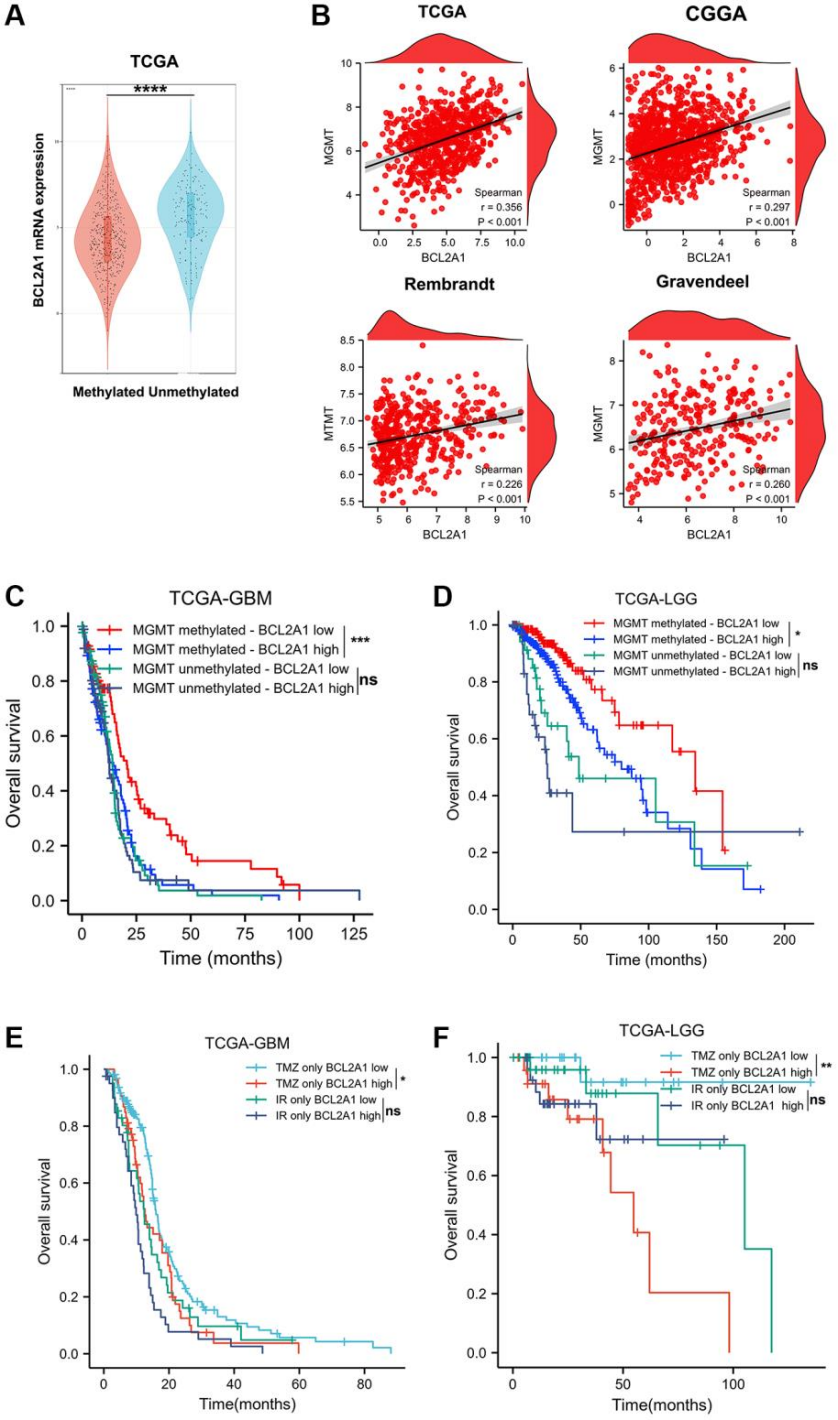
**BCL2A1 was an independent predictor of response to temozolomide in gliomas.** (**A**) BCL2A1 expression in gliomas with methylated or unmethylated MGMT promoters. (**B**) The correlation between BCL2A1 and MGMT expression in public datasets. (**C**, **D**) Effect of BCL2A1 on the prognosis of patients with GBM and LGG with different MGMT promoter methylation statuses. (**E**, **F**) Effect of BCL2A1 on the prognosis of glioma patients who received TMZ chemotherapy or IR alone. Abbreviations: IR: ion radiotherapy; ns: non-significant. ^*^P < 0.05, ^**^P < 0.01. ^***^P < 0.001, ^****^P < 0.0001.

Temozolomide (TMZ) is a clinical first-line chemotherapy drug that is used to treat glioma patients. Although TMZ treatment improves the prognosis of glioma patients, resistance to TMZ is inevitable in most patients [24]. The methylation of O6-methylguanine methyltransferase (MGMT) is a repair protein that can induce chemotherapy resistance by repairing DNA damage caused by TMZ, and MGMT promoter methylation can inhibit MGMT protein expression and enhance the sensitivity of glioma patients to TMZ treatment [25]. Therefore, we investigated whether BCL2A1 affects resistance to TMZ chemotherapy in gliomas. We first investigated the relationship between MGMT promoter methylation status and BCL2A1 expression in the TCGA database. The results showed that high BCL2A1 expression was associated with unmethylated MGMT promoter status (Figure 10A). In addition, correlation analysis demonstrated that BCL2A1 was positively correlated with expression in the TCGA, CGGA, Rembrandt and Gravendeel datasets (Figure 10B). These results suggested that BCL2A1 may influence the therapeutic responsiveness of glioma patients to TMZ. Interestingly, we divided patients into groups according to MGMT promoter methylation status, and survival analysis showed that high expression of BCL2A1 predicted poor prognosis in GBM and LGG patients with MGMT promoter methylation. However, BCL2A1 expression did not accurately predict the survival of glioma patients with unmethylated MGMT promoters (Figure 10C, 10D). For glioma patients treated with TMZ alone, BCL2A1 expression accurately predicted the survival of GBM and LGG patients. However, for glioma patients who received only radiotherapy (IR), the expression of BCL2A1 did not affect their prognosis (Figure 10E, 10F). In summary, these data demonstrated that BCL2A1 was an independent predictor of response to TMZ chemotherapy in gliomas.

In addition, we explored the relationship between BCL2A1 expression and sensitivity to the top 12 anticancer drugs from the CellMiner database. The data indicated that high BCL2A1 expression could increase the drug IC50 and decrease the drug sensitivity of vemurafenib (selective oral inhibitor of the BRAF V600 kinase), dabrafenib (reversible inhibitor of mutant BRAF kinase), hypothemycin, PD-98059 (inhibitor of ERK1/2), selumetinib (selective inhibitor of MAPK kinase), bafetinib (tyrosine kinase inhibitor), cobimetinib (MEK inhibitor), ABT-199 (BCL-2 inhibitor), trametinib (MEK inhibitor), okadaic acid (inhibitor of protein phosphatase type 1) and ixazomib citrate (inhibitor of 20 S proteasome β5), and high BCL2A1 expression decreased the drug IC50 and increased the drug sensitivity of pyrazoloacridine (inhibitor of topoisomerases 1 and 2) (Supplementary Figure 2).

### Knockdown of BCL2A1 inhibited GBM cell proliferation and macrophage migration

**Figure 11:**
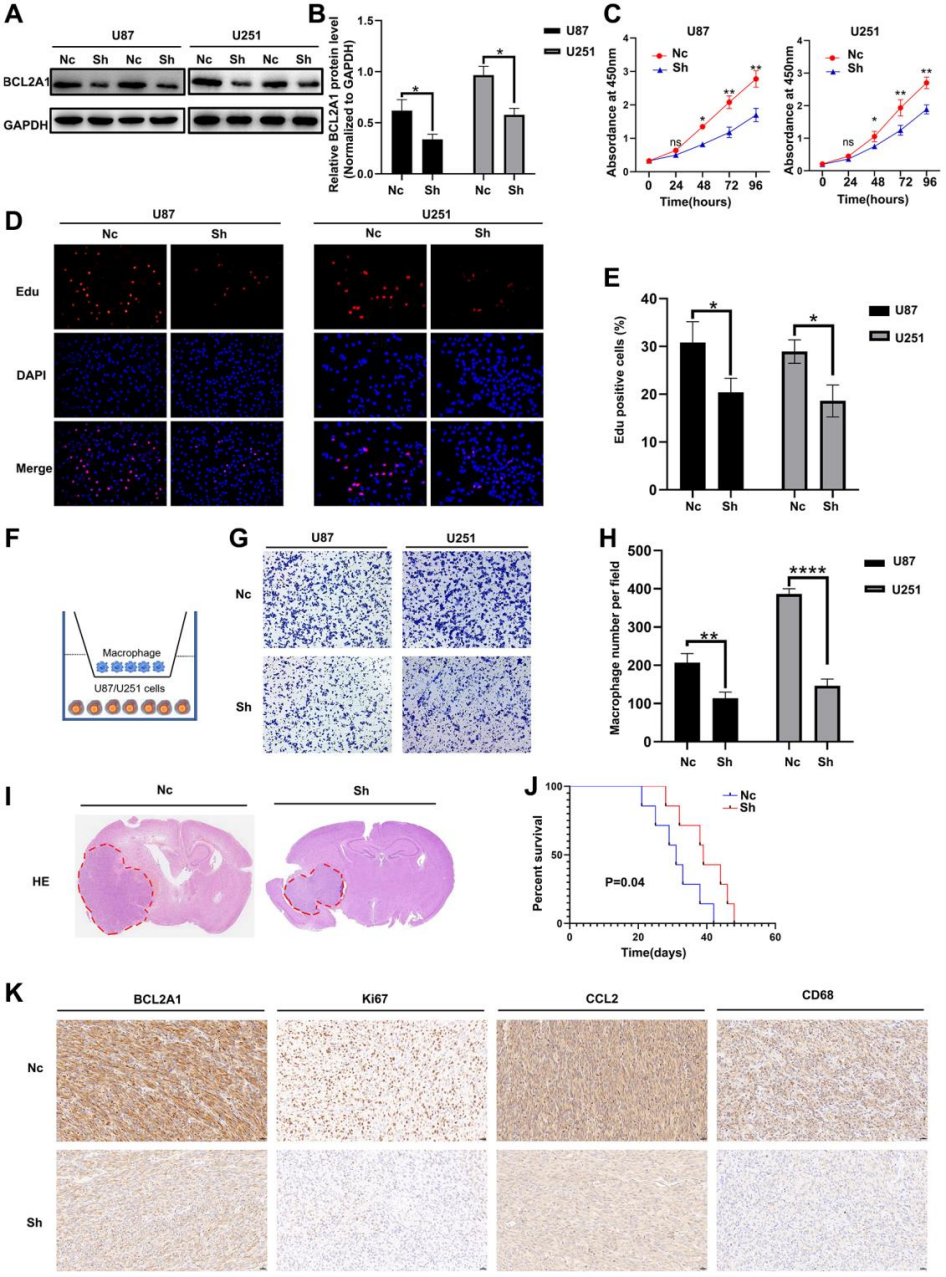
**Knockdown of BCL2A1 inhibited GBM cell proliferation and macrophage migration.** (**A**, **B**) Western blot analysis of the knockdown effect of BCL2A1 in U87 and U251 cells. (**C**) The effect of BCL2A1 knockdown on proliferation of GBM cells was detected by CCK8. (**D**, **E**) Edu assay indicated that BCL2A1 knockdown inhibited proliferation of GBM cells. (**F**) Diagram of co-culture of macrophages and GBM cells. (**G**, **H**) Knockdown of BCL2A1 in GBM cells inhibited the migration ability of co-cultured macrophages. (**I**) HE staining analysis. (**J**) Analysis of Kaplan-Meier survival in mice. (**K**) IHC analysis of BCL2A1, Ki67, CD68 and CCL2. ns: non-significant; ^*^P < 0.05, ^**^P < 0.01. Abbreviations: Nc: control group; Sh: BCL2A1 knockdown group.

To further verify the biological function of BCL2A1 in GBM, we used ShRNA to specifically knockdown the expression of BCL2A1. As shown in Figure 11A, 11B, western blot indicated that BCL2A1 expression was obviously knocked down in U87 and U251 cells (Supplementary Figure 3). In addition, CCK8 and Edu assay were employed to detect the effects of BCL2A1 expression on GBM cell proliferation. The results showed that the proliferation rate of GBM cells in the BCL2A1 knockdown group was slower than that in the control group (Figure 11C), and the Edu cell positive rate was also lower than that in the control group (Figure 11D, 11E). We also tested whether BCL2A1 could medicate cell proliferation using an intracranial xenograft model *in vivo*. As we suspected, mice implanted with BCL2A1 knockdown cells produced smaller tumor volumes than controls (Figure 11I), and Kaplan-Meier analysis demonstrated that the BCL2A1 knockdown group mice have a longer survival time than control group mice (Figure 11J). Furthermore, IHC staining showed that the number of KI67 positive cells in BCL2A1 knockdown group was significantly less than that in control group (Figure 11K). To further verify whether BCL2A1 is involved in the regulation of macrophage migration, we used PMA to induce THP-1 cells into macrophages. Subsequently, we used a Transwell device o co-culture macrophage with U87 and U251 cells (Figure 11F). As expected, knockdown of BCL2A1 in U87 and U251 cells inhibited the migration ability of co-cultured macrophages (Figure 11G, 11H). Moreover, mice in the BCL2A1 knockdown group were found to have lower expression of CD68 and CCL2 *in vivo* models (Figure 11K).

## DISCUSSION

Due to the high heterogeneity of glioma, there are great differences between individual patients. Therefore, the treatment of glioma should be comprehensively considered according to individual prognostic factors, clinical symptoms and tumor progression. Genetic tests can be used to guide treatment. For example, patients with MGMT promoter methylation are more sensitive to TMZ chemotherapy, while patients with 1p19 codeletion are considered not to receive radiotherapy [26]. To overcome the limitations of the current standard treatment for glioma, scientists are working to develop new treatment strategies. Immunotherapy is considered a potential treatment for glioma. However, tumor-infiltrating immune cells in the tumor microenvironment can regulate the immune escape of tumor cells and affect the therapeutic effect of tumors [27]. There is still a lack of reliable markers for the early diagnosis of glioma and the assessment of immunotherapy response.

As a member of the antiapoptotic BCL2 family, BCL2A1 is associated with the occurrence, progression and drug resistance of various tumors. However, the expression pattern and potential biological function of BCL2A1 in gliomas are still unknown. In this study, we found that the expression of BCL2A1 in glioma was significantly higher than that in NBTs at both the mRNA and protein levels according to a comprehensive analysis of public databases and an in-house cohort that included 10 NBTs and 174 glioma samples. Moreover, the expression of BCL2A1 is correlated with the WHO grade and malignancy of glioma, and its high expression reduces the survival time of patients with LGG and GBM. These results suggest that BCL2A1 may be a potential prognostic marker in gliomas.

In addition, GO and KEGG analyses showed that BCL2A1 may be involved in the immune response and immune-related biological processes, including cytokine-cytokine receptor interactions, natural killer cell-mediated cytotoxicity, the IL17 signaling pathway, and the Toll-like receptor (TLR) signaling pathway. IL-17 can mediate the recruitment of specific γδ T-cell subpopulations and activate the PI3K/Akt1/NF-κB-p65 pathway to promote glioma progression [3]. The tumor microenvironment is a complex system composed of tumor cells, infiltrating lymphocytes, immune cells, fibroblasts and endothelial cells, and it is closely related to the occurrence, malignant progression and metastasis of tumors [27]. We found that the expression of BCL2A1 is closely related to immune, stromal, and ESTIMATE scores, suggesting that BCL2A1 plays an important role in the immune microenvironment of glioma. We also found that BCL2A1 is positively correlated with most immune checkpoint genes, suggesting that BCL2A1 may induce immune escape by participating in the regulation of immune checkpoint genes, thus promoting tumorigenesis.

Subsequently, we investigated the association of BCL2A1 immune cells using the TIMER 2.0 database and single-cell sequencing data. The results showed that BCL2A1 was significantly associated with mononuclear/macrophage infiltration in glioma. In addition, there is growing evidence that TAMs play a key role in tumor cell progression and metastasis and are highly represented in the glioma immune microenvironment [28].

Correlation analysis indicated that BCL2A1 was positively correlated with TAM markers in public datasets. To further validate our findings, we performed IHC and multiple immunofluorescences staining on two of the TAM markers and found that the expression of BCL2A1 was significantly correlated with CCL2 and CD68, and they were coexpressed in gliomas. In addition, we demonstrated *in vitro* that inhibition of BCL2A1 expression weakened the migration ability of co-cultured macrophages, and *in vivo* models also found that knockdown of BCL2A1 was closely related to low expression of CD68 and CCL2. We found that BCL2A1 was positively correlated with chemokines and chemokine receptors. Chemokines secreted by tumors play an important role in the regulation of macrophage differentiation and the tumor immune microenvironment. CCL2 is a member of the chemokine family, which can promote tumor immune escape and cancer cell proliferation through recruitment of TAMs [29]. Therefore, we speculate that BCL2A1 may promote TAM infiltration in glioma by influencing chemokines, including CCL2, and affect the tumor microenvironment and tumor progression in glioma. Our study found that BCL2A1 was mainly enriched in the mesenchymal subtype of GBM. Previous evidence suggests that mesenchymal GBM has higher TAM infiltration than other subtypes, and TAMs may contribute to tumor progression and metastasis by affecting epithelial-mesenchymal transition (EMT) [30, 31]. We also demonstrated that knocking down BCL2A1 inhibits proliferation of GBM cells *in vivo* and *in vitro*, while extending survival time in mice. These results reveal a potential mechanism underlying the poor prognosis in patients with high BCL2A1 expression.

TMZ is a first-line treatment for glioma, and the MGMT promoter methylation status is a factor that predicts therapeutic responses to TMZ. However, the accuracy of MGMT promoter methylation in predicting responses to TMZ is limited [32]. Our study found that glioma patients with high BCL2A1 expression had poor responses to TMZ treatment. BCL2A1 may be used as a supplementary marker to predict the response of glioma patients to TMZ chemotherapy. BCL2A1 has also been associated with resistance to chemotherapy in breast cancer, melanoma and colon cancer [11, 33, 34]. The reason for resistance to chemotherapy may be that BCL2A1 can inhibit the apoptosis induced by chemotherapy drugs. The hypoxia-induced M2 phenotype of TAMs promotes cell proliferation and TMZ resistance in GBM cells by activating the PI3K/Akt/Nrf2 pathway [35]. In another study, TAMs increased CCL2 secretion through M2 polarization and activated the PI3K/Akt/mTOR signaling pathway in breast cancer cells, promoting endocrine resistance [36]. Therefore, we speculate that BCL2A1 may promote the resistance of glioma to TMZ chemotherapy by regulating the infiltration of TAMs in the immune tumor microenvironment.

## CONCLUSION

In summary, our results suggested that BCL2A1 was an independent prognostic marker and potential predictor of sensitivity to TMZ chemotherapy in glioma patients. Moreover, overexpression of BCL2A1 was closely related to TAM infiltration in the glioma immune microenvironment. These results provide insights into the cellular and molecular basis of the glioma tumor immune microenvironment and identify new targets for glioma immunotherapy.

## Supplementary Materials

Supplementary Figures

Supplementary Tables
