# Supplementary material for: BCL2A1 is associated with tumor-associated macrophages and unfavorable prognosis in human gliomas
Source: Aging (Albany NY). 2023 Oct 25;15(20):11611–38. doi: 10.18632/aging.205149 (PMC10637801; doi:10.18632/aging.205149)
Supplement: Supplementary Figures [file aging-15-205149-s001.pdf]

SUPPLEMENTARY FIGURES

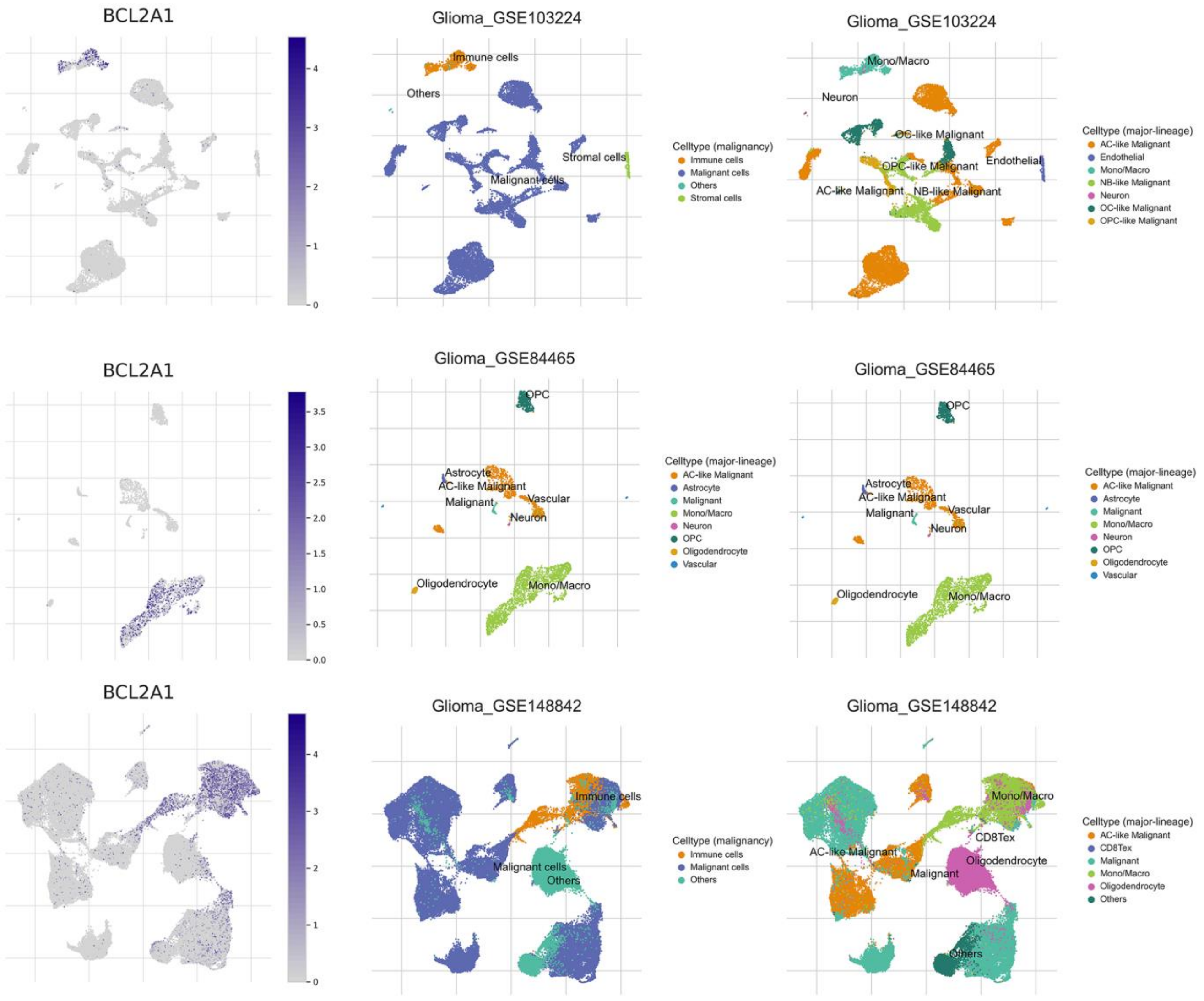

Supplementary Figure 1. BCL2A1 expression was associated with macrophages in TISCH 2.0 database.

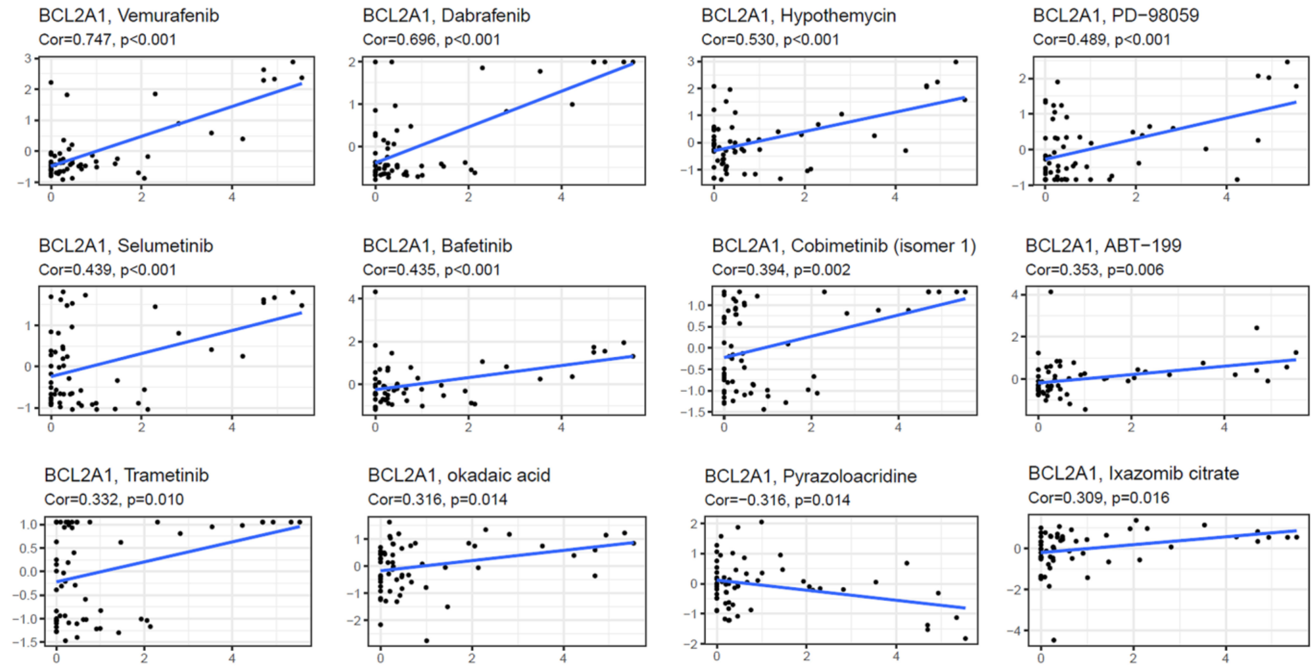

Supplementary Figure 2. Drug sensitivity analysis of BCL2A1 based on CellMiner database.

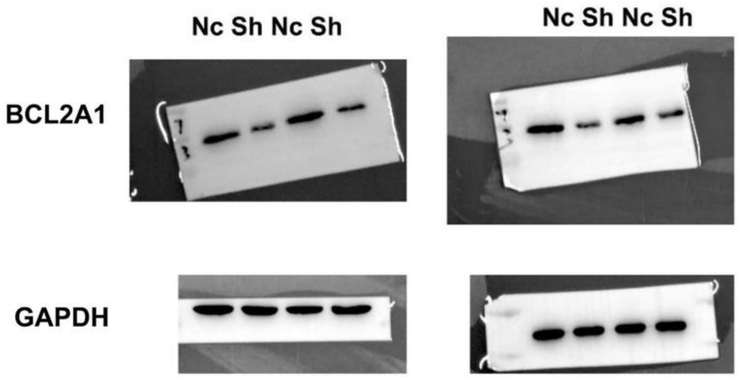

Supplementary Figure 3. Original WB blots.
