# Supplementary material for: BCL2A1 is associated with tumor-associated macrophages and unfavorable prognosis in human gliomas
Source: Aging (Albany NY). 2023 Oct 25;15(20):11611–38. doi: 10.18632/aging.205149 (PMC10637801; doi:10.18632/aging.205149)
Supplement: Supplementary Tables [file aging-15-205149-s002.pdf]

SUPPLEMENTARY TABLES

Supplementary Table 1. Correlation between BCL2A1 and clinicopathological characteristics in patients with gliomas in CGGA.

Clinicopathological characteristics	BCL2A1 expression		*P* value
	Low (*n* = 489)	High (*n* = 490)	
**Age**			
≥60	35	73	<0.001
<60	453	412	
**Gender**			
Male	279	295	>0.05
Female	210	195	
**WHO grade**			
II–III	379	226	<0.001
IV	110	264	
**IDH status**			
Mutant	306	206	<0.001
Wild-type	150	268	
**Chr.1p19q**			
Codeletion	149	56	<0.001
Non-codeletion	272	431	
**Chemotherapy**			
Yes	283	335	<0.001
No	158	113	
**Radiotherapy**			
Yes	373	386	<0.05
No	94	62	

Supplementary Table 2. GO in GBM by GSEA analysis.

Description	ES	NES	*P*-value
GOBP_ADAPTIVE_IMMUNE_RESPONSE	0.723	2.391	0.014
GOBP_RESPONSE_TO_CHEMOKINE	0.82	2.267	0.017
GOBP_NEUTROPHIL_CHEMOTAXIS	0.808	2.252	0.016
GOBP_LEUKOCYTE_PROLIFERATION	0.699	2.249	0.014
GOBP_LYMPHOCYTE_MEDIATED_IMMUNITY	0.721	2.248	0.015
GOBP_LEUKOCYTE_CELL_CELL_ADHESION	0.69	2.241	0.014
GOMF_CHEMOKINE_RECEPTOR_BINDING	0.835	2.217	0.017
GOBP_REGULATION_OF_T_CELL_ACTIVATION	0.685	2.215	0.014
GOBP_CELLULAR_RESPONSE_TO_BIOTIC_STIMULUS	0.722	2.212	0.016
GOMF_CCR_CHEMOKINE_RECEPTOR_BINDING	0.854	2.212	0.017
GOBP_NEUTROPHIL_MIGRATION	0.797	2.201	0.016
GOBP_POSITIVE_REGULATION_OF_LEUKOCYTE_CELL_CELL_ADHESION	0.706	2.186	0.016
GOCC_SPECIFIC_GRANULE	0.75	2.182	0.017
GOBP_POSITIVE_REGULATION_OF_CELL_ACTIVATION	0.668	2.176	0.014
GOBP_REGULATION_OF_LEUKOCYTE_PROLIFERATION	0.711	2.175	0.016
GOMF_CHEMOKINE_ACTIVITY	0.841	2.162	0.017
GOBP_NEGATIVE_REGULATION_OF_IMMUNE_SYSTEM_PROCESS	0.657	2.161	0.014

GOBP_T_CELL_MIGRATION	0.813	2.161	0.017
GOBP_T_CELL_ACTIVATION	0.663	2.158	0.014
GOBP_RESPONSE_TO_MOLECULE_OF_BACTERIAL_ORIGIN	0.663	2.157	0.014
GOBP_POSITIVE_REGULATION_OF_T_CELL_PROLIFERATION	0.774	2.155	0.016
GOBP_CELLULAR_RESPONSE_TO_MOLECULE_OF_BACTERIAL_ORIGIN	0.739	2.154	0.018
GOBP_REGULATION_OF_ADAPTIVE_IMMUNE_RESPONSE	0.728	2.151	0.016
GOBP_REGULATION_OF_IMMUNE_EFFECTOR_PROCESS	0.657	2.15	0.014
GOBP_GRANULOCYTE_MIGRATION	0.748	2.143	0.016
GOBP_T_CELL_PROLIFERATION	0.736	2.142	0.017
GOBP_REGULATION_OF_LYMPHOCYTE_ACTIVATION	0.653	2.142	0.014
GOBP_POSITIVE_REGULATION_OF_CYTOKINE_PRODUCTION	0.649	2.141	0.014
GOBP_LYMPHOCYTE_MIGRATION	0.767	2.141	0.016
GOBP_MONONUCLEAR_CELL_DIFFERENTIATION	0.648	2.136	0.014
GOBP_REGULATION_OF_LEUKOCYTE_MEDIATED_IMMUNITY	0.71	2.131	0.017
GOBP_REGULATION_OF_LYMPHOCYTE_MEDIATED_IMMUNITY	0.73	2.122	0.016
GOBP_POSITIVE_REGULATION_OF_DEFENSE_RESPONSE	0.648	2.107	0.014
GOMF_CYTOKINE_RECEPTOR_BINDING	0.67	2.104	0.014
GOCC_TERTIARY_GRANULE	0.72	2.102	0.017
GOBP_NEGATIVE_REGULATION_OF_LYMPHOCYTE_MEDIATED_IMMUNITY	0.805	2.098	0.017
GOBP_LYMPHOCYTE_CHEMOTAXIS	0.799	2.089	0.017
GOBP_NEGATIVE_REGULATION_OF_LEUKOCYTE_MEDIATED_IMMUNITY	0.804	2.084	0.018
GOBP_POSITIVE_REGULATION_OF_CELL_CELL_ADHESION	0.665	2.083	0.014
GOBP_CELL_CHEMOTAXIS	0.657	2.082	0.014

Supplementary Table 3. GO in LGG by GSEA analysis.

Description	ES	NES	*P*-value
GOBP_POSITIVE_REGULATION_OF_LEUKOCYTE_CELL_CELL_ADHESION	0.752	1.96	0.011
GOBP_POSITIVE_REGULATION_OF_CELL_ACTIVATION	0.731	1.956	0.01
GOBP_ADAPTIVE_IMMUNE_RESPONSE	0.719	1.941	0.01
GOBP_GRANULOCYTE_CHEMOTAXIS	0.775	1.937	0.011
GOBP_NEUTROPHIL_CHEMOTAXIS	0.792	1.935	0.011
GOBP_ALPHA_BETA_T_CELL_ACTIVATION	0.756	1.934	0.011
GOBP_POSITIVE_REGULATION_OF_CELL_CELL_ADHESION	0.733	1.931	0.011
GOBP_NEUTROPHIL_MIGRATION	0.771	1.929	0.011
GOBP_REGULATION_OF_T_CELL_ACTIVATION	0.718	1.919	0.01
GOBP_GRANULOCYTE_MIGRATION	0.754	1.918	0.011
GOBP_T_CELL_PROLIFERATION	0.74	1.91	0.011
GOBP_RESPONSE_TO_CHEMOKINE	0.783	1.906	0.011
GOBP_LYMPHOCYTE_MIGRATION	0.763	1.902	0.011
GOBP_LYMPHOCYTE_MEDIATED_IMMUNITY	0.718	1.894	0.01
GOBP_REGULATION_OF_IMMUNE_EFFECTOR_PROCESS	0.7	1.893	0.01
GOBP_REGULATION_OF_ADAPTIVE_IMMUNE_RESPONSE	0.736	1.889	0.011
GOBP_REGULATION_OF_LEUKOCYTE_MEDIATED_IMMUNITY	0.726	1.888	0.011
GOBP_REGULATION_OF_LYMPHOCYTE_ACTIVATION	0.701	1.888	0.01
GOBP_LEUKOCYTE_CELL_CELL_ADHESION	0.7	1.885	0.01
GOBP_REGULATION_OF_LYMPHOCYTE_MEDIATED_IMMUNITY	0.733	1.876	0.011
GOBP_POSITIVE_REGULATION_OF_T_CELL_PROLIFERATION	0.769	1.875	0.011

GOBP_REGULATION_OF_ALPHA_BETA_T_CELL_ACTIVATION	0.768	1.875	0.011
GOBP_REGULATION_OF_LEUKOCYTE_MEDIATED_CYTOTOXICITY	0.776	1.872	0.012
GOBP_REGULATION_OF_CELL_KILLING	0.767	1.869	0.011
GOBP_REGULATION_OF_LEUKOCYTE_PROLIFERATION	0.716	1.864	0.011
GOBP_T_CELL_ACTIVATION	0.69	1.855	0.01
GOBP_CD4_POSITIVE_ALPHA_BETA_T_CELL_DIFFERENTIATION	0.762	1.853	0.012
GOBP_ALPHA_BETA_T_CELL_DIFFERENTIATION	0.753	1.852	0.011
GOBP_RESPONSE_TO_INTERFERON_GAMMA	0.717	1.851	0.011
GOBP_T_CELL_DIFFERENTIATION_INVOLVED_IN_IMMUNE_RESPONSE	0.776	1.848	0.013
GOBP_POSITIVE_REGULATION_OF_CYTOKINE_PRODUCTION	0.683	1.847	0.01
GOBP_POSITIVE_REGULATION_OF_LEUKOCYTE_MEDIATED_IMMUNITY	0.732	1.843	0.011
GOMF_CHEMOKINE_ACTIVITY	0.827	1.843	0.014
GOBP_B_CELL_MEDIATED_IMMUNITY	0.735	1.837	0.011
GOBP_LEUKOCYTE_MEDIATED_CYTOTOXICITY	0.741	1.835	0.011
GOBP_T_CELL_DIFFERENTIATION	0.7	1.835	0.011
GOCC_TRANSCRIPTIONALLY_ACTIVE_CHROMATIN	0.898	1.833	0.014
GOBP_RESPONSE_TO_INTERLEUKIN_1	0.709	1.833	0.011
GOBP_POSITIVE_REGULATION_OF_LEUKOCYTE_PROLIFERATION	0.718	1.833	0.011
GOMF_IMMUNE_RECEPTOR_ACTIVITY	0.733	1.827	0.011

Supplementary Table 4. KEGG in GBM by GSEA analysis.

Description	ES	NES	*P*-value
KEGG_CYTOKINE_CYTOKINE_RECEPTOR_INTERACTION	0.719	2.192	0.014
KEGG_NOD_LIKE_RECEPTOR_SIGNALING_PATHWAY	0.815	2.1	0.016
KEGG_CHEMOKINE_SIGNALING_PATHWAY	0.706	2.068	0.015
KEGG_HEMATOPOIETIC_CELL_LINEAGE	0.725	1.889	0.015
KEGG_TOLL_LIKE_RECEPTOR_SIGNALING_PATHWAY	0.698	1.849	0.016
KEGG_INTESTINAL_IMMUNE_NETWORK_FOR_IGA_PRODUCTION	0.738	1.833	0.016
KEGG_LEISHMANIA_INFECTION	0.702	1.828	0.015
KEGG_GRAFT_VERSUS_HOST_DISEASE	0.788	1.815	0.018
KEGG_NATURAL_KILLER_CELL_MEDIATED_CYTOTOXICITY	0.645	1.808	0.016
KEGG_CYTOSOLIC_DNA_SENSING_PATHWAY	0.699	1.756	0.016
KEGG_TYPE_I_DIABETES_MELLITUS	0.743	1.741	0.018
KEGG_ALLOGRAFT_REJECTION	0.756	1.712	0.018
KEGG_CELL_ADHESION_MOLECULES_CAMS	0.609	1.709	0.016
KEGG_PRIMARY_IMMUNODEFICIENCY	0.749	1.695	0.018
KEGG_JAK_STAT_SIGNALING_PATHWAY	0.598	1.694	0.015
KEGG_T_CELL_RECEPTOR_SIGNALING_PATHWAY	0.627	1.67	0.016
KEGG_AUTOIMMUNE_THYROID_DISEASE	0.664	1.63	0.017
KEGG_B_CELL_RECEPTOR_SIGNALING_PATHWAY	0.618	1.582	0.016
KEGG_RIG_I_LIKE_RECEPTOR_SIGNALING_PATHWAY	0.613	1.574	0.015
KEGG_TASTE_TRANSDUCTION	−0.689	−1.808	0.024
KEGG_COMPLEMENT_AND_COAGULATION_CASCADES	0.602	1.568	0.03
KEGG_RIBOSOME	0.597	1.553	0.031
KEGG_OLFACTORY_TRANSDUCTION	0.431	1.345	0.043

Supplementary Table 5. KEGG in LGG by GSEA analysis.

Description	ES	NES	*P*-value
KEGG_CYTOKINE_CYTOKINE_RECEPTOR_INTERACTION	0.729	1.96	0.01
KEGG_CHEMOKINE_SIGNALING_PATHWAY	0.716	1.898	0.011
KEGG_HEMATOPOIETIC_CELL_LINEAGE	0.751	1.857	0.012
KEGG_SYSTEMIC_LUPUS_ERYTHEMATOSUS	0.694	1.805	0.011
KEGG_LEISHMANIA_INFECTION	0.746	1.8	0.012
KEGG_T_CELL_RECEPTOR_SIGNALING_PATHWAY	0.694	1.794	0.011
KEGG_GRAFT_VERSUS_HOST_DISEASE	0.836	1.792	0.014
KEGG_NOD_LIKE_RECEPTOR_SIGNALING_PATHWAY	0.751	1.79	0.013
KEGG_INTESTINAL_IMMUNE_NETWORK_FOR_IGA_PRODUCTION	0.787	1.765	0.013
KEGG_ALLOGRAFT_REJECTION	0.826	1.758	0.014
KEGG_PRIMARY_IMMUNODEFICIENCY	0.817	1.74	0.014
KEGG_COMPLEMENT_AND_COAGULATION_CASCADES	0.718	1.733	0.012
KEGG_JAK_STAT_SIGNALING_PATHWAY	0.655	1.732	0.011
KEGG_ASTHMA	0.819	1.722	0.014
KEGG_CELL_ADHESION_MOLECULES_CAMS	0.659	1.716	0.011
KEGG_TYPE_I_DIABETES_MELLITUS	0.762	1.701	0.013
KEGG_PORPHYRIN_AND_CHLOROPHYLL_METABOLISM	0.768	1.688	0.013
KEGG_VIRAL_MYOCARDITIS	0.689	1.66	0.012
KEGG_PENTOSE_AND_GLUCURONATE_INTERCONVERSIONS	0.781	1.641	0.014
KEGG_NATURAL_KILLER_CELL_MEDIATED_CYTOTOXICITY	0.628	1.635	0.011
KEGG_TOLL_LIKE_RECEPTOR_SIGNALING_PATHWAY	0.635	1.61	0.012
KEGG_B_CELL_RECEPTOR_SIGNALING_PATHWAY	0.653	1.607	0.011
KEGG_DRUG_METABOLISM_OTHER_ENZYMES	0.684	1.577	0.013
KEGG_AUTOIMMUNE_THYROID_DISEASE	0.675	1.571	0.013
KEGG_FC_EPSILON_RI_SIGNALING_PATHWAY	0.621	1.528	0.012
KEGG_STARCH_AND_SUCROSE_METABOLISM	0.663	1.506	0.013
KEGG_APOPTOSIS	0.597	1.496	0.012
KEGG_PRION_DISEASES	0.698	1.487	0.014
KEGG_ANTIGEN_PROCESSING_AND_PRESENTATION	0.597	1.479	0.012
KEGG_ECM_RECEPTOR_INTERACTION	0.571	1.421	0.012
KEGG_OLFACTORY_TRANSDUCTION	0.513	1.38	0.01
KEGG_ASCORBATE_AND_ALDARATE_METABOLISM	0.809	1.66	0.014
KEGG_FOCAL_ADHESION	0.497	1.321	0.021
KEGG_STEROID_HORMONE_BIOSYNTHESIS	0.623	1.45	0.025
KEGG_CYTOSOLIC_DNA_SENSING_PATHWAY	0.605	1.423	0.025
KEGG_BLADDER_CANCER	0.609	1.374	0.038
KEGG_LEUKOCYTE_TRANSENDOTHELIAL_MIGRATION	0.523	1.349	0.046
KEGG_AMYOTROPHIC_LATERAL_SCLEROSIS_ALS	0.425	1.284	0.045
